# Ion channel/Stat6-driven nano-immune programming of tissue-resident macrophages by amide-functionalized nanocellulose

**DOI:** 10.1016/j.bioactmat.2026.03.038

**Published:** 2026-04-09

**Authors:** Sayan Deb Dutta, Jeong Man An, Jagannath Mondal, Subhankar Bose, Santosh Kumar Jana, Youjin Seol, Chandan Kuman Maity, Yong-kyu Lee, Ki-Taek Lim

**Affiliations:** a Department of Biosystems Engineering, Kangwon National University, Chuncheon, 24341, Gangwon-do, Republic of Korea; b Institute of Forest Science, Kangwon National University, Chuncheon, 24341, Gangwon-do, Republic of Korea; c School of Medicine, University of California Davis, Sacramento, 95817, California, United States; d 4D Convergence Technology Institute (National Key Technology Institute in University), Korea National University of Transportation, Chungju, 27469, Republic of Korea; e Future Innovative Materials Global Leading Research Center, Korea National University of Transportation, Chungju, 27469, Republic of Korea; f Department of Green Bio Engineering, Korea National University of Transportation, Chungju, 27470, Republic of Korea; g Cancer Biology and Inflammatory Disorder Division, CSIR-Indian Institute of Chemical Biology, Kolkata, 700032, West Bengal, India; h Department of Microbiology, University of Calcutta, Kolkata, 700019, West Bengal, India; i Interdisciplinary Program in Smart Agriculture, Kangwon National University, Chuncheon, 24341, Gangwon-do, Republic of Korea; j Department of Chemistry, Gachon University, Sujeong-gu, Seongnam-si, Gyeonggi-do, 13120, Republic of Korea; k Department of Chemical and Biological Engineering, Korea National University of Transportation, Chungju, 27470, Republic of Korea

**Keywords:** Functionalized nanocellulose, Nano-immune interaction, Surface chemistry, Macrophage fate, Tissue engineering

## Abstract

Nanocellulose has long been studied as a bioactive material for tissue engineering; however, the mechanisms underlying its surface chemistry-mediated immune reprogramming remain unclear. Herein, we report a comprehensive multi-omics study of pristine cellulose nanocrystals (CNCs) and amide-functionalized CNCs (a-CNCs) to elucidate their '*nano-immune*' interaction and impact on tissue-resident macrophages *in vivo*. Using integrated scRNA-Seq, bulk RNA-Seq, pharmacological inhibition, and histological profiling, we reveal that a-CNCs exhibit outstanding biocompatibility, showing no pro-inflammatory activation of macrophages across major organs within 14-day subacute window. In particular, a-CNCs exposure correlates with enhanced voltage-gated ion channel (*KCa3.1* and *Scn1b*) and *Stat6* signaling, while suppressing *Nfkb*-driven pro-inflammatory signals. This suggest that ion channel activation is strongly associated with M2 macrophage polarization. Moreover, a 28-day splenocytes profiling revealed no observable increase in CD4^+^/CD8^+^ T cells, suggesting non-adaptive immune response after a-CNC exposure. Concurrently, pseudotime mapping further discloses that a-CNC exposure preserves natural macrophage developmental trajectories across organ niches, while pristine CNCs induce mild M1-skewing in the spleen. *In vitro* validation confirms that a-CNCs intrinsically drive a pro-healing phenotype in macrophages, underscoring that macro-scale immune behavior can be transcriptionally triggered through nano-level surface chemistry of CNCs.

## Highlights

• Amide-functionalized nanocellulose enables long-term immune-safe interaction with tissue-resident macrophages *in vivo*.
• Single-cell transcriptomics reveal preserved macrophage developmental trajectories without inflammatory perturbation.
• Surface chemistry driven M2 macrophage polarization via Stat6 and voltage-gated ion channel (KCa3.1/Scn1b) activation.
• Pristine CNCs induce mild, organ-specific M1 skewing, while a-CNCs suppress NF-κB–mediated pro-inflammatory signaling.
• Nano-level surface chemistry regulates immune behavior, redefining nanocellulose as an immuno-instructive biomaterial.

## Graphical abstract

**Figure:**
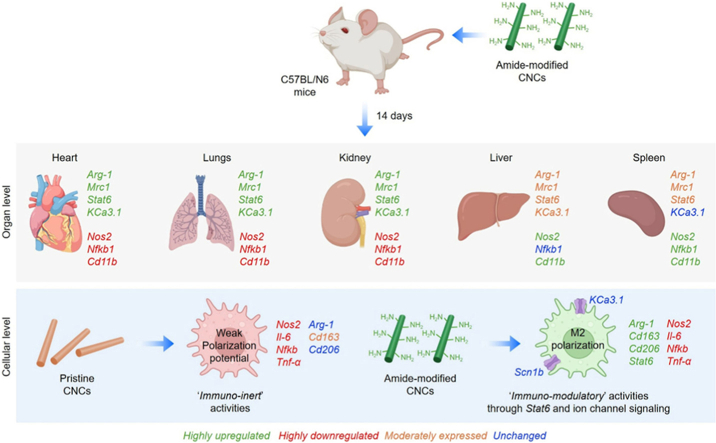

## 1 Introduction

Macrophages, a type of immune cell, are key orchestrators of host-biomaterial interactions, coupling pathogen defense during tissue repair and regeneration. The ability to steer macrophage phenotype between pro-inflammatory (M1, classically activated, pathogen killing phenotype) and anti-inflammatory (M2, alternately activated, tissue repair phenotype) states has therefore emerged as a key design principle for immunomodulatory materials [1]. Nanocellulose, a hydrolysis product of cellulose, has long been studied as an attractive nanomaterial in biomedical engineering owing to its tailorable size, surface chemistry, and high aspect ratio, making it ideal for drug delivery, bioimaging, and biosensing applications [[2], a), b)].

Recent studies demonstrate that nanocellulose can be engineered to either facilitate macrophage activation or polarization, while maintaining biosafety [3]. For instance, early toxicology studies revealed that pristine cellulose nanofibrils (CNFs) and nanocrystals (CNCs) displayed low cytotoxicity in lung normal fibroblast (MRC-5) cells. In contrast, carboxylic or sulfated CNCs activated the transcription factors *Tnf-α and Il-1β*, leading to M1 macrophage polarization [4]. Colić and co-workers reported that pristine CNCs can trigger inflammasome-mediated signaling during M1 polarization, which is largely dependent on particle size, aspect ratio, and functionalization [5]. Moreover, Patel et al. examined the role of CNCs' shape (*e*.*g*., spherical or rod-shaped) on macrophage polarization. They concluded that culture time profoundly affected macrophage polarization towards the M1 phenotype, beyond its surface topography [3]. Similarly, rod-shaped CNCs with hydroxyl (–OH), carboxylic (–COOH), and/or sulfhydryl (–SO_3_H) moieties and variable size (*e*.*g*., ∼150-720 nm) have been shown to polarize alveolar macrophages towards M1 phenotype via activation of *Il-1β*, *Nos2*, and *Tnf-α* through NOD-LRR-pyrin domain-containing protein 3 (NLRP3) inflammasome and clathrin-mediated pathways [a), b)]. Besides, positively charged CNCs (*e*.*g*., cationic or peptide-modified) and bacterial nanocellulose (BNC) have been shown to suppress *Tnf-α* expression while upregulating M2 markers, such as *Cd206* and *Arg-1*, in tumor-associated macrophages (TAMs) and human acute monocytic leukemia (THP-1) cells *in vitro* [[7], a), b)]. Heilala and co-workers also reported that phosphate-modified CNF gel induces M2a (*Il-4* or *Il-13* induced alternately activated) macrophage polarization by upregulating the expression of *Cd206*, *Il-4*, and *Il-10* [8]. These reports clearly suggest that macrophage polarization depends on surface charge and/or functional groups rather than on shape or size. Furthermore, several reports demonstrate that orally delivered CNCs are minimally absorbed and largely eliminated by the gastrointestinal tract, whereas intravenous injection results in a transient accumulation in the liver and spleen [9]. Although several reports indicate dynamic switching between M1/M2 states after application of CNCs and/or CNC-based biomaterial scaffolds *in vitro* and/or *in vivo*, the long-term fate, genotoxicity, cardiotoxicity, surface chemistry of CNCs and its effects on tissue-resident macrophages, and underlying mechanisms have not been elucidated to date.

**Scheme 1:**
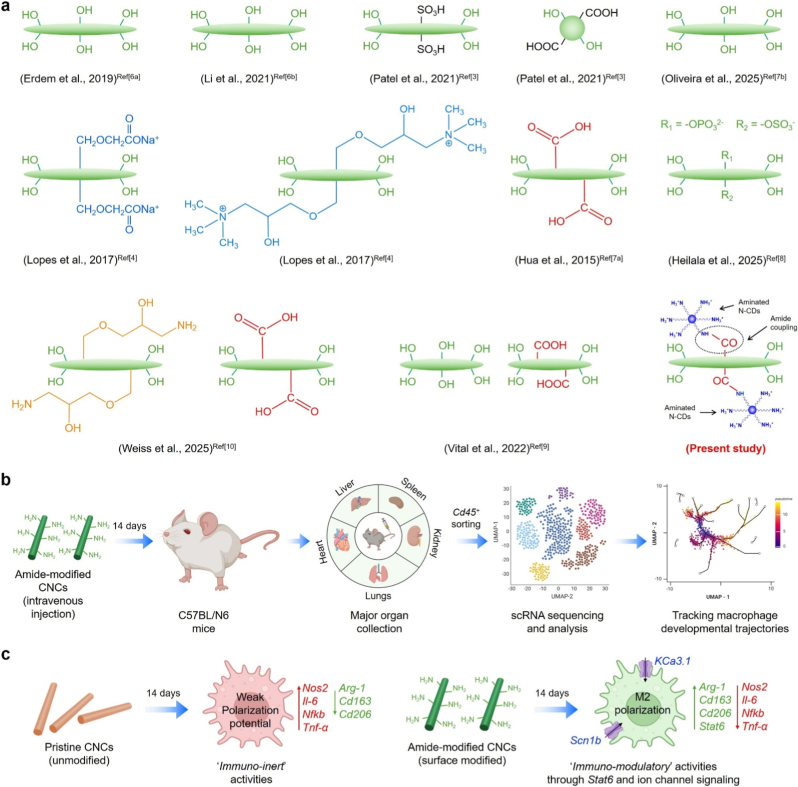
**(a)** Representative surface chemistries of cellulose nanocrystals (CNCs) previously reported in the literature. The present study introduces an amide-functionalized CNC generated by covalent coupling of amine-rich carbon dots (N-CDs), producing a surface enriched in amine functionalities. **(b)** Schematic of the *in vivo* experimental workflow. **(c)** Comparative immunomodulatory behaviors of pristine vs. amide-functionalized CNCs reported in this study.

To investigate the surface chemistry-mediated immune reprogramming, we fabricated two CNC samples, *i*.*e*., pristine (CNCs with surface –OH, and –SO_3_H groups, length: ∼140 nm) and amide-modified CNCs (CNCs with surface –OH, –COOH, and –NH/NH_2_ groups, length: ∼140 nm, a-CNCs). The uptake of pristine CNCs and a-CNCs was studied in RAW 264.7 murine macrophage cells *in vitro*, while *in vivo* uptake, biocompatibility, and macrophage fate determination were assessed in C57BL/6 mice. A schematic illustration of the scope of our present study is shown in Scheme 1. The surface amide decoration transforms CNCs from an ‘*immuno-neutral*’ to an ‘*immuno-instructive*’ material by activating ion channels in macrophages. The ‘*nano-immune*’ interaction was investigated using single-cell RNA sequencing, histopathology, and immunocytochemistry. We identified key differentially expressed genes (DEGs) upon a-CNCs intravenous treatment that are associated with tissue-resident macrophages, mainly localized in TLF^+^ (*Cd163*^hi+^, *Lyve1*^hi+^, *Timd4*^hi+^, and *Folr2*^hi+^) cell clusters, as revealed by pseudotime analysis, suggesting precursor-like states toward mature tissue-resident identities up to 14 days. The spleen- and liver-resident macrophages showed a slight upregulation of pro-inflammatory phenotypes compared to heart, lung, and kidney-resident macrophages. Moreover, the bulk RNA sequencing study identified several DEGs strongly associated with upregulation of cationic ion channel activity (*KCa3.1* and *Scn1b*) and *Stat6* signaling. In contrast, a downregulation of the nuclear factor kappa-beta (*Nfkb*) signaling pathway was observed following a-CNCs exposure in RAW 264.7 cells at day 14. Notably, an ion channel inhibition study using various pharmacological inhibitors (Nifedipine, TRAM-34, and AS1810722) further revealed deactivation of ion channel activity with a downregulation of M2 phenotype, highlighting the pro-healing and regenerative role of a-CNCs. We also validated the single-cell and bulk sequencing data by qRT-PCR and immunostaining, confirming the activation of a pro-healing phenotype at day 14. Collectively, the multi-organ transcriptomic analysis demonstrates that a-CNCs exert no detectable acute or pro-inflammatory signatures at the cellular, transcriptional, or functional levels, underscoring their strong immunocompatibility for future biomedical applications.

## 2 Results and discussion

### 2.1 Characterization and biosafety evaluation of functionalized nanocellulose

**Fig. 1:**
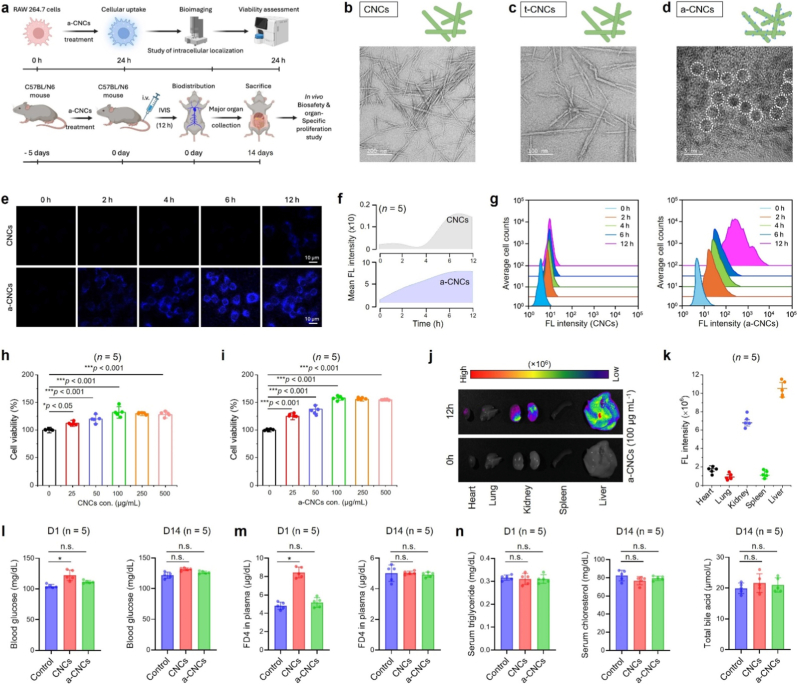
*In vitro* and *in vivo* biocompatibility studies of the pure CNCs and a-CNCs. **(a)** Schematic illustration of the biosafety assessment strategies of the fabricated samples. **(b**–**d)** Representative HR-TEM images with corresponding diagrams of the pure CNCs, t-CNCs, and a-CNCs. Scale bar: 5, 100, and 200 nm. **(e, f)** Representative CLSM images of the RAW 264.7 cells with corresponding mean FL intensities showing the uptake of CNCs and a-CNCs (100 μg mL^−1^ each) at the indicated time points. Scale bar: 10 μm (*n* = 5 each). **(g)** Flow cytometry analysis of the cellular uptake of CNCs and a-CNCs using RAW 264.7 cells *in vitro* (*n* = 5 each). **(h, i)** WST-8 assay results showing the viability of RAW 264.7 cells after 24 h incubation with various concentrations (0-500 μg mL^−1^) of CNCs and a-CNCs (*n* = 5 each). **(j, k)***In vivo* biodistribution analysis with FL intensities of major organs (*e.g*., heart, lung, kidney, spleen, and liver) extracted after 12 h post-administration of a-CNCs (100 μg mL^−1^/10 g B W., *n* = 5). **(l)** Intraperitoneal glucose tolerance test of mice after 2 weeks' post-administration (*n* = 5 each). **(m)** Gut integrity test (FD4 plasma concentration) of the mice after 2 weeks' post-administration (*n* = 5 each). **(n)** Representative serum triglyceride, cholesterol, and total bile acid estimation from blood collected after 2 weeks' post-administration. Data reported as mean ± s.d. of replicated experiments (*n* = 5 each), statistical significance was considered at ∗*p* < 0.05 and ∗∗∗*p* < 0.001 (One-way ANOVA test with Tukey's test *post hoc* analysis).

Fig. 1 schematically illustrates the *in vitro* and *in vivo* biosafety evaluation of pristine CNCs and surface-engineered CNCs. The detailed synthesis and functionalization of CNCs to a-CNCs is given in Fig. S1(a). The crystallinity indices for CNCs, t-CNCs, and a-CNCs were calculated to be ∼66.67%, ∼64.13%, and ∼60.29%, respectively, suggesting that surface functionalization did not alter the intrinsic structure [10]. High-resolution TEM (HR-TEM) imaging confirms that the chemical modification preserves the intrinsic rod-like morphology of CNCs while introducing nanoscale surface roughness and a more homogeneous dispersion profile (Fig. 1(b–d)), indicative of successful surface functionalization. Furthermore, the HR-TEM images of a-CNCs reveal that N-CDs appear on the surface of CNCs with a crystal spacing of 0.21 nm (crystallite size: ∼4.34 nm), indicating successful grafting (Fig. S1(b)). The particle size of both CNCs (length: ∼141.15 ± 8.87 nm; width: ∼9.32 ± 1.21 nm) and a-CNCs (length: ∼142.85 ± 3.34 nm; width: ∼8.99 ± 2.35 nm) remains same before and after modification, while the N-CDs particle size was measured to be around ∼3.65 ± 0.81 nm (Fig. S1(c)). This was also reflected in their surface potential and the abundance of surface carboxylic groups (–COO^-^). As depicted in Fig. S1(d), the zeta potential (ζ) was gradually decreased when we moved from pristine CNCs → t-CNCs → a-CNCs. The change in zeta potential was attributed to the presence of amine-functionalized N-CDs on the surface of CNCs [10,11]. Strikingly, the carboxylic group density onto the surface of t-CNCs (2.14 ± 0.15 mmol cm^−2^) was significantly decreased in a-CNCs (0.61 ± 0.13 mmol cm^−2^), further validating the adsorption and bonding of N-CDs onto the CNCs surface (Fig. S1(e)).

The structural and functional changes in pristine CNCs and a-CNCs were also investigated using X-ray diffraction (XRD), Fourier transform infrared (FT-IR), and X-ray photoelectron spectroscopy (XPS). As shown in Fig. S1(f), the XRD spectra of the pristine CNCs revealed characteristic peaks at 2θ = 21.56° and 23.91°, corresponding to the (200) and (004) of typical Cellulose-I domains. Interestingly, a-CNCs showed a slight reduction in the (200) peak intensity, suggesting minor disruption of the cellulose crystalline domain by surface grafting. Similarly, the FT-IR spectra provide molecular confirmation of the chemical change (Fig. S1(g)), with a-CNCs showing typical absorption bands for –NH, –NH_2_, and –COOH originating from the grafted N-CDs. Moreover, high-resolution XPS analysis confirms the chemical composition of pristine CNCs and a-CNCs. The C1s XPS spectra show a remarkable enhancement in C-N and C=O bonds in a-CNCs after N-CDs grafting (Fig. S1(h and i)). The N1s XPS spectra of a-CNCs suggest the appearance of distinct peaks at 398.3 eV and 400 eV, corresponding to the –NH and –NH_2_/C-N, confirming the presence of nitrogenous moieties on the a-CNC surface (Fig. S1(j and k)) [10,12]. These results confirmed that a-CNC surface is mostly composed of –NH, –NH_2_, and –COOH groups.

*In vitro* cellular internalization studies using murine macrophages (RAW 264.7) reveal a time-dependent increase in fluorescence intensity (0-12 h) for both materials (Fig. 1(e and f)), with a-CNCs exhibiting markedly enhanced intracellular accumulation. This was further confirmed by flow cytometry analysis, which also verified enhanced uptake and fluorescence of a-CNCs (Fig. 1(g)). This improved uptake is attributed to their tailored surface chemistry (*e*.*g*., presence of –NH_2_ and –COO^-^ groups), which facilitates more efficient membrane interaction and endocytic transport [10,13]. Cytocompatibility was systematically quantified using water soluble tetrazolium-8 (WST-8) assays, demonstrating that both CNCs and a-CNCs exhibit negligible cytotoxicity across a broad concentration range (0-500 μg mL^−1^) with a higher viability at 100 μg mL^−1^ concentration after 24 h (Fig. 1(h and i)). Cell viability remained consistently >95%, confirming that surface modification of CNCs does not induce adverse cellular responses.

This was also reflected in the *in vivo* uptake study in C57BL/N6 mice. The *in vivo* biodistribution analysis shows that intravenously administered a-CNCs preferentially accumulate in the liver and spleen, organs commonly associated with reticuloendothelial system (RES) clearance, without detectable off-target accumulation or acute toxicity (Fig. 1(j and k)). Moreover, the metabolic and physiological parameters, such as glucose tolerance test (GTT), gut integrity assay (GIA) using fluorescein isothiocyanate-dextran-4 (FD4) leakage, revealed non-significant alteration of glucose metabolism and epithelial barrier function in a-CNC treated group at day 14 (Fig. 1(l and m)), suggesting its biocompatibility [14]. Serum biochemical markers, such as triglycerides, cholesterol, and total bile acid content, remained unchanged at day 14, indicating that a-CNCs exposure does not hinder lipid homeostasis or hepatic function *in vivo* (Fig. 1(n)). To validate this, we evaluated organ-level cytotoxicity and routine blood profiles at day 14 post-administration of CNCs and a-CNCs. As shown in Fig. S2(a), histology of major organs showed no inflammation, tissue damage, or structural anomalies in CNCs and a-CNCs-treated mice, suggesting their organ-protective nature. Besides, hematological parameters, such as red blood cell (RBC) indices, white blood cell (WBC) differentials, and platelet counts, remained within the normal physiological range, confirming the absence of systemic toxicity and immune disruption at day 14 (Fig. S2(b–k)), which was quite similar to previously reported findings [[15], a), b)]. Collectively, these findings underscore that a-CNCs maintain excellent biocompatibility, enhanced cellular uptake, superior RES clearance, and no detectable systemic toxicity, making them a highly promising nanomaterial for therapeutic applications.

### 2.2 *In vivo* organ-specific immunomodulation studies

Next, we investigated the organ-specific potential for macrophage polarization of pristine CNCs and a-CNCs after 14 days of administration. Prior to all *in vivo* studies, we treated C57BL/6 mice with clodronate liposomes to deplete F4/80^+^ macrophages, a common immunobiological technique [16]. The details of the macrophage depletion experiment are shown in Fig. S3(a). The flow cytometry analysis confirmed a dramatic dose-dependent reduction in F4/80^+^ macrophages in the spleen (Fig. S3(b and c)) after the first and second doses of liposome treatment, and this was validated by immunocytochemical staining. As shown in Fig. S3(d and e), the first and second doses of liposome significantly (∗∗∗∗*p* < 0.0001) reduced the F4/80^+^ macrophage in the spleen, suggesting a successful depletion. This was also verified by measuring levels of inflammatory cytokines, such as TNF-α, IL-1β, IL-6, and IFN-γ, after the second liposome dose. As depicted in Fig. S3(f–i), the serum cytokine levels of TNF-α, IL-1β, IL-6, and IFN-γ were remarkably decreased compared to the control group, suggesting that macrophage depletion changed immune homeostasis, which is an ideal model for drug/biomolecule testing [17].

**Fig. 2:**
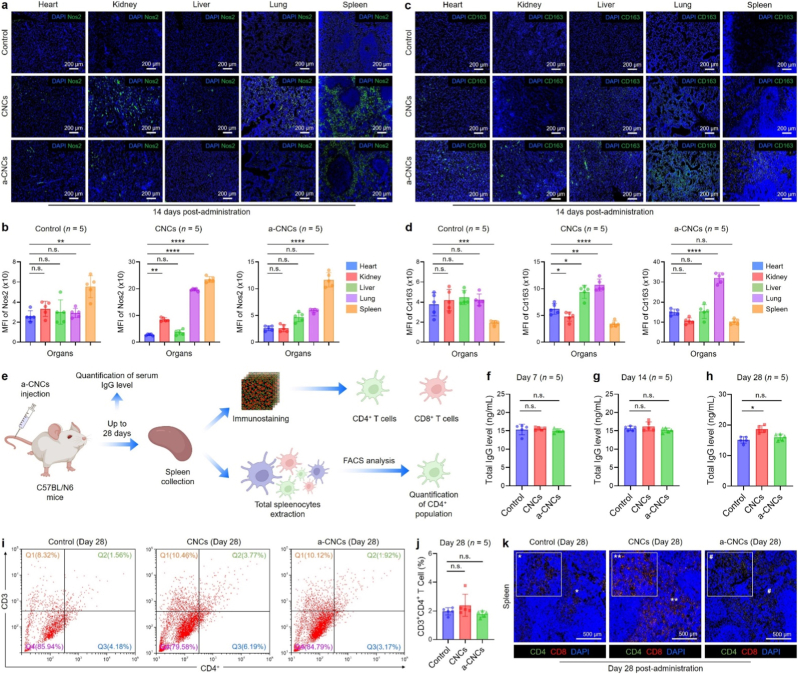
*In vivo* tissue-specific macrophage staining for M1/M2 markers after CNCs and a-CNCs administration at day 14. **(a, c)** Representative immunostaining images of major organs (heart, kidney, liver, lung, and spleen) showing the expression of Nos2 and Cd163 markers at day 14 (*n* = 5 each). Scale bar: 200 μm. **(b, d)** Statistical analysis of mean fluorescence intensity (MFI) of Nos2 and Cd163 across all organs (*n* = 5). **(e)** Schematic illustration of the long-term cellular immunity *in vivo*. **(f**–**h)** Representative serum IgG level after various treatment up to day 28 (*n* = 5 each). **(i, j)** FACS analysis of CD4^+^ T cell population after immunized with PBS (control), pristine CNCs (100 μg mL^−1^) and a-CNCs (100 μg mL^−1^) at day 28 (*n* = 5 each). **(k)** Immunostaining of spleen showing the expression of CD4^+^ (green) and CD8^+^ (red) cells at day 28 post-administration. Scale bar: 500 μm (*n* = 5 each). Data reported as mean ± s.d. of replicated experiments, statistical significance was considered at ∗*p* < 0.05, ∗∗*p* < 0.01, ∗∗∗*p* < 0.001, and ∗∗∗∗*p* < 0.0001 (One-way ANOVA test with Tukey's test *post hoc* analysis).

Fig. 2 depicts the tissue-level immunostaining of macrophages after exposure to CNCs and a-CNCs for 14 days. Immunofluorescence staining for the M1 protein marker, Nos2, revealed minimal basal expression in control tissues, with only sparse Nos2^+^ macrophages distributed in the liver and spleen, organs naturally enriched in phagocytes (Fig. 2(a and b)). As discussed earlier, the intravenous administration led to preferential accumulation of CNCs and a-CNCs in liver and spleen, key components of the mononuclear phagocyte system (MPS) where high densities of phagocytic Kupffer cells and splenic red pulp macrophages govern nanoparticle clearance. These tissue-resident macrophages are ontogenetically and transcriptionally distinct from macrophages in heart, lung, and kidney, exhibiting specialized metabolic programs, scavenger (*e*.*g*., reactive oxygen species) receptor expression, and cytokine responsiveness [[18], a), b)]. The pristine CNC-treated mice showed a mild, organ-dependent increase in Nos2 signal, particularly in the spleen and lung, consistent with transient macrophage activation associated with RES clearance. In contrast, a-CNC-treated tissues displayed markedly reduced Nos2 fluorescence intensity relative to the CNC group, with most organs showing near-baseline levels comparable to those of untreated controls. In addition, Cd163 showed an interesting pattern of expression. Control groups showed moderate Cd163^+^ staining in the liver and spleen, reflecting the natural abundance of tissue-resident M2-like macrophages (Kupffer cells and red pulp macrophages) [5]. Interestingly, a-CNCs treatment resulted in a significant enhancement of Cd163^+^ macrophages in multiple organs, including the liver, kidney, lung, and spleen, indicating a systemic shift toward a pro-healing, anti-inflammatory activation (Fig. 2(c and d)). This increase in Cd163 expression coincided with the reduced Nos2 staining observed in the same tissues, highlighting a coordinated bias toward M2-dominant polarization. Such a phenotype is consistent with an immune microenvironment that favors tissue protection, inflammation resolution, and extracellular matrix remodeling—properties desirable for regenerative and therapeutic biomaterials [8,19]. Moreover, we also investigated the potential adaptive immune response following systemic exposure of a-CNCs up to 28 days through serum IgG level and spleen immune profiling (Fig. 2(e)). The quantitative serum immunoglobulin-gamma (IgG) profile suggested no significant alteration of IgG level when we move from day 7 to day 28 following CNCs and a-CNCs exposure (Fig. 2(f–h)). Concurrently, Flow cytometry analysis demonstrated that the frequencies of total CD3^+^ T cells, as well as CD4^+^ helper and CD8^+^ cytotoxic T-cell subsets, remained statistically indistinguishable from controls at days 7, 14, and 28 (Fig. 2(i and j)). Importantly, the CD4^+^/CD8^+^ ratio, an established indicator of immune homeostasis and antigen-driven T-cell maturation remain preserved across all groups, suggesting the absence of systemic immune activation or immunosuppressive imbalance. This was further confirmed by immunostaining of spleen tissue against CD4/CD8 markers at day 28. As shown in Fig. 2(k), pristine CNC-treated groups exhibited a slightly higher expression for CD8, while no noticeable change for CD4/CD8 was observed for a-CNCs when compared with control group. The quantification analysis is shown in Fig. S4. These results demonstrate that a-CNC administration does not induce stress, macrophage-driven chronic inflammation, or adaptive immune dysregulation within the 14-day subacute window, with preserved T-cell homeostasis up to 28 days.

### 2.3 Single-cell transcriptomics reveal localization-specific polarization of tissue-resident macrophages

**Fig. 3:**
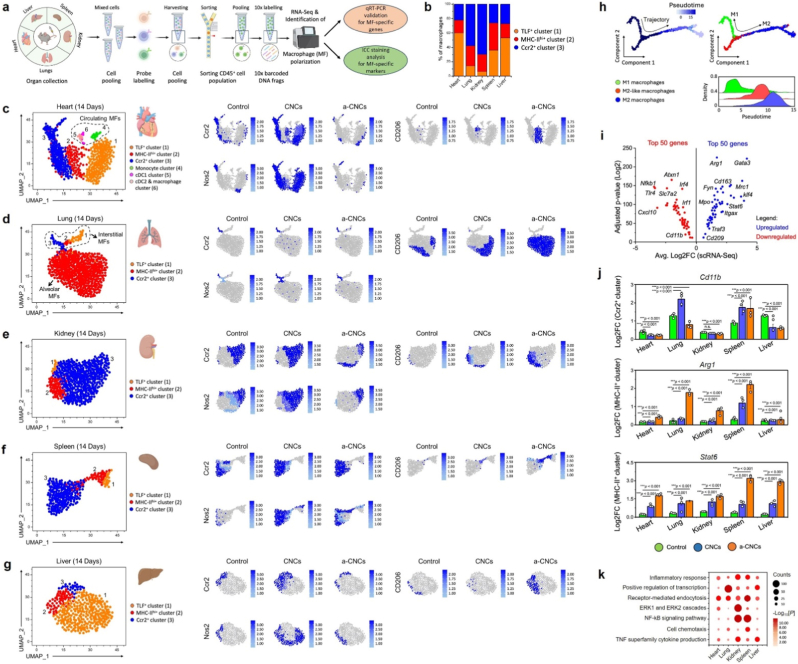
scRNA-Seq revealed localization-dependent polarization of tissue-resident macrophages *in vivo*. **(a)** Schematic illustration of the library preparation and scRNA-Seq analysis from major organs (*e.g*., heart, lung, kidney, spleen, and liver) after 14 days of administration. **(b)** Representative bar graphs showing the percentage of TLF^+^, MHC-II^+^, and Ccr2^+^ macrophages in various organs, as obtained from *CellRanger*. **(c**–**g)** UMAP projections of macrophage populations isolated from heart, lung, kidney, spleen, and liver on day 14, showing distinct cluster identities and expression profiles of Ccr2, Nos2, and Cd206 markers in control, pristine CNCs, and a-CNCs groups. **(h)** Pseudotime trajectory analysis demonstrating macrophage states across various samples. **(i)** Volcano plot showing the expression of the top 50 upregulated and downregulated genes when comparing the a-CNCs *vs*. CNCs groups. **(j)** Expression changes (Log2FC, ∗*p* < 0.05) of key polarization markers in Ccr2^+^ and MHC-II^+^ clusters across all organs (*n* = 3 each). **(k)** Gene ontology (GO) enrichment analysis showing the significant pathways elevated in biological process (BP) when comparing a-CNCs *vs*. CNCs at day 14.

To gain insights into system-wide macrophage polarization at transcriptional level after CNCs and a-CNCs administration, we used single-cell RNA-Seq (scRNA-Seq) to examine macrophage identity, heterogeneity, and functional expression across major organs *in vivo* within 14-day subacute window. Fig. 3(a) schematically depicts the study pipeline and a high-resolution view of potential *nano-immune* interactions between pristine CNCs and a-CNCs after 14 days’ post-administration. The scRNA-Seq analysis was performed after extracting resident macrophages from the heart, lung, kidney, spleen, and liver using well-established protocols. After quality control (QC) and filtering, the raw data obtained for 159,789 cells were further analyzed, with cells having a median gene count of 1468 genes/cell. We sorted macrophages/monocytes using a broad macrophage gating strategy (CD45^+^CD64^+hi^), a common strategy for macrophage subtype identification [20]. In our combined study of all five organs, Louvain clustering yielded 18,380 single-cell transcriptomes across 17 populations. Out of them, 5 were identified as macrophage lineages based on *Adgre1*^+^, *C1qb*^+^, *C1qc*^+^, *Fcgr1*^+^, and *Cd14*^+^ genes, while the remaining 12 were identified as macrophage-monocyte interfaces, monocytes, lymphocytes, dendritic cells, and other circulating cells, respectively.

To examine the macrophage sub-clusters and their gene expression levels within each organ, we separately sub-clustered them for each organ using an unbiased *Unified Manifold Approximation and Projection* (UMAP)-based clustering. For this, we excluded monocytes (= undifferentiated cells), dendritic cells (CD1C^+^), lymphocytes (CD4^+^ and CD8^+^), monocyte-T cell doublets, and other circulating cells to improve interpretation. A shown in Fig. 3(b), the macrophage atlas reveals 3 major macrophage subtypes: TLF^+^ cluster (typically tissue-resident and shows distinct self-renewal potential; high in *Cd163*^+^, *Lyve1*^+^, *Timd4*^+^, and *Folr2*^+^), major histocompatibility complex-II or MHC-II^hi^ cluster (transitional lipid-processing macrophages; *MHC-II*^hi+^, *Cd52*^hi+^, *Ccr2*^+^, *S100*^+^, *Cd163*^-^, *Lyve1*^-^, *Timd4*^-^, and *Folr2*^-^), and Ccr2^+^ cluster (cytokine-regulating macrophages, *Lyve1*^-^, *Timd4*^-^, and *Folr2*^-^, *Ccr2*^hi+^, *Il-1β*^+^, *Il-6*^+^, and *Tnf-α*^+^). The MHC-II^+^ and Ccr2^+^ cell clusters are typically involved in angiogenesis, tissue remodeling, and wound healing. In contrast, the TLF^+^ cell clusters are mainly associated with anti-inflammatory activation, ferroptosis, and fibrosis [20,21]. It was interesting to note that the relative proportions of these macrophage clusters remained remarkably stable between control, CNCs, and a-CNCs-treated groups across all organs at day 14. This indicates that neither CNCs nor a-CNCs disrupt macrophage population or their plasticity, an essential hallmark of immune biocompatibility.

Moreover, when mapped onto organ-specific UMAP plots (Fig. 1(c–g)), the macrophages displayed organ-specific spatial distributions, with no emergence of abnormal inflammatory foci after pristine CNC or a-CNC exposure up to 14 days. In the case of heart-resident macrophages, the control and a-CNC-treated groups exhibited less abundance of Ccr2^+^ and Nos2^+^ macrophages (∼5-10%). In comparison, a slightly higher density (∼48%) was observed in the pristine CNC-treated group (Fig. 3(c)). Similarly, a greater abundance of CD206^+^ macrophages was found in the a-CNC-treated group (>80%) compared to the control or CNC-treated groups. The lung and kidney-resident macrophages showed a similar trend of abundance, suggesting no apparent pro-inflammatory activation over 14 days (Fig. 3(d and e)). Strikingly, the spleen-resident macrophages showed a dramatic increase in Ccr2^+^ and Nos2^+^ macrophages (∼45-56%) with a lesser abundance of CD206^+^ macrophages (∼15%), suggesting toxicity and pro-inflammatory activation (Fig. 3(f)). This was owing to greater accumulation of CNCs and the mononuclear phagocytic system (MPS), which recognize and engulf various nanoparticles and nanomaterials during metabolism [[22], b), a)]. Besides, the liver-resident macrophages also revealed a low abundance of Ccr2^+^ (∼18%), Nos2^+^ (∼12-20%), and CD206^+^ (∼11-25%) macrophages owing to the rapid RES clearance (Fig. 3(g)). Collectively, these results suggest that CNCs had a slight toxic effect on the spleen, whereas the a-CNC did not cause macrophage relocation, accumulation, or niche disruption, indicating no immunological stress response. This was also correlated with the pseudotime trajectories of the macrophage subtypes. The pseudotime analysis demonstrated a uniform developmental trajectory across the three macrophage clusters (*e*.*g*., M1, M2-like, and M2 macrophages), progressing smoothly from precursor-like states toward mature tissue-resident identities (Fig. 3(h)). Importantly, pseudotime densities for CNC- and a-CNC-treated groups completely overlapped with those of controls (∗*p* < 0.05), showing that the CNC-based material does not accelerate, delay, or redirect macrophage differentiation. This highlights that the CNCs (with or without surface functionalization) are transcriptionally inert with respect to macrophage development.

Next, we examined the top 100 differentially expressed genes (DEGs; Log2FC, ∗*p* < 0.05) in the TLF^+^ cluster that are actively involved in macrophage plasticity and reprogramming in a-CNCs *vs*. CNCs across all organs using a volcano plot. As shown in Fig. 3(i), among the top 100 DEGs, 50 genes were found upregulated, while 50 genes were found highly downregulated in all the organs at day 14 post-administration. Among them, a comparison between a-CNCs *vs*. CNCs uncovered 19 genes (*Atxn1*, *Nfkb1*, *Tlr4*, *Irf4*, *Slc7a2*, *Irf1*, *Cxcl10*, and *Cd11b*, Arg*1*, *Gata3*, *Cd163*, *Fyn*, *Mrc1*, *Mpo*, *Klf4*, *Stat6*, *Itgax4*, *Traf3*, and *Cd209*), which were found differentially (Log2FC, ∗*p* < 0.05) regulated across all the organs. A detailed, organ-specific expression profile of 19 key DEGs is shown in Fig. S5(a). Notably, several M1 phenotype-associated genes (*e*.*g*., *Nfkb1*, *Tlr4*, *Cxcl10*, and *Slc7a2*) were significantly down-regulated, while key M2 phenotype-associated genes (*e*.*g*., Arg*1*, *Trf4*, *Cd163*, *Mrc1*, and *Stat6*) were upregulated, suggesting a shift towards pro-healing and anti-inflammatory macrophage differentiation [23]. A STRING protein-protein network (Fig. S5(b)) and k-means clustering analysis (Fig. S5(c–e)) further identified the two major functional gene clusters enriched with '*activation of IL-4/IL-10 signaling*' and suppression of '*interferon-mediated inflammatory signaling*', which was verified by KEGG_BP (Fig. S5(f)) and REAC_tissue expression (Fig. S5(g)) databases. This remarkable finding suggests the activation of both pro-inflammatory (∼19-22%) and anti-inflammatory (∼65-70%) pathways with no signs of stress-response or apoptosis-related transcripts [24], with anti-inflammatory macrophages dominant.

Moreover, the expression quantification of representative functional genes in Ccr2^+^ (*Cd11b*) and MHC-II^+^ (Arg*1* and *Stat6*) clusters further revealed the activation of M2-like resident macrophages over M1 macrophages across all organs at 14-day subacute window. While the expression of *Cd11b* remained unchanged across all organs, we detected significantly higher expression of Arg1 and Stat6 following a-CNCs treatment (Fig. 3(j)), underscoring its pro-healing and non-inflammatory nature [3,25]. Furthermore, a KEGG pathway enrichment analysis showed that specific inflammatory pathways, such as '*inflammatory response*,' '*TNF signaling*,' and '*NF-kB signaling pathway,*' were not significantly enriched across all organs when compared between a-CNCs *vs*. CNCs (Fig. 3(k)), confirming the immune safety and pro-healing nature of the a-CNCs. Moreover, to evaluate the role of a-CNCs on adaptive immune response, we performed scRNA-Seq of splenocytes harvested at day 14 post-administration (Fig. S6(a)). Unsupervised clustering identified major immune populations, including T cells, NK cells, B cells, antigen-presenting cells (APCs), neutrophils, monocytes, and macrophages. Comparative compositional analysis revealed only modest changes in immune cell proportions between control and a-CNC groups, indicating preserved global immune architecture (Fig. S6(b and c). Within the T-cell cluster (C_1), differential transcriptional profiling demonstrated condition-dependent modulation of effector-associated programs. The heatmaps showed minimally altered expression patterns in CD4^+^ and CD8^+^ T-cell subsets in both control and a-CNC group (Fig. S6(d)). Furthermore, the UMAP projection plots identified key cytotoxic and lineage-specific genes, which were not significantly increased after a-CNCs treatment when compared to control (Fig. S6(e)). Chromatin accessibility-based transcription factor enrichment analysis indicated slightly higher enrichment scores for Eomes binding motifs in cytotoxic and effector memory T-cell subsets relative to naïve and regulatory populations (Fig. S6(f)). Besides, violin plots further confirmed baseline regulation of effector genes and memory T cell-associated markers after a-CNCs treatment, suggesting a mild shift towards activated/effector-memory phenotype (Fig. S6(g)). This suggests that a-CNCs treatment slightly enriched the cytotoxic and Eomes-associated phenotypes within the CD8^+^ T cell population, while preservation of naïve and regulatory signatures argues against systemic immune hyperactivation. Taken together, the multi-organ single-cell transcriptomic analysis demonstrates that a-CNCs exert no detectable immune or inflammatory changes at the cellular, transcriptional, or functional pathway levels within the 14-day subacute window, suggesting they are exceptional biocompatible for clinical applications.

### 2.4 Bulk RNA-Seq analysis reveals M2 macrophage polarization upon a-CNC uptake

**Fig. 4:**
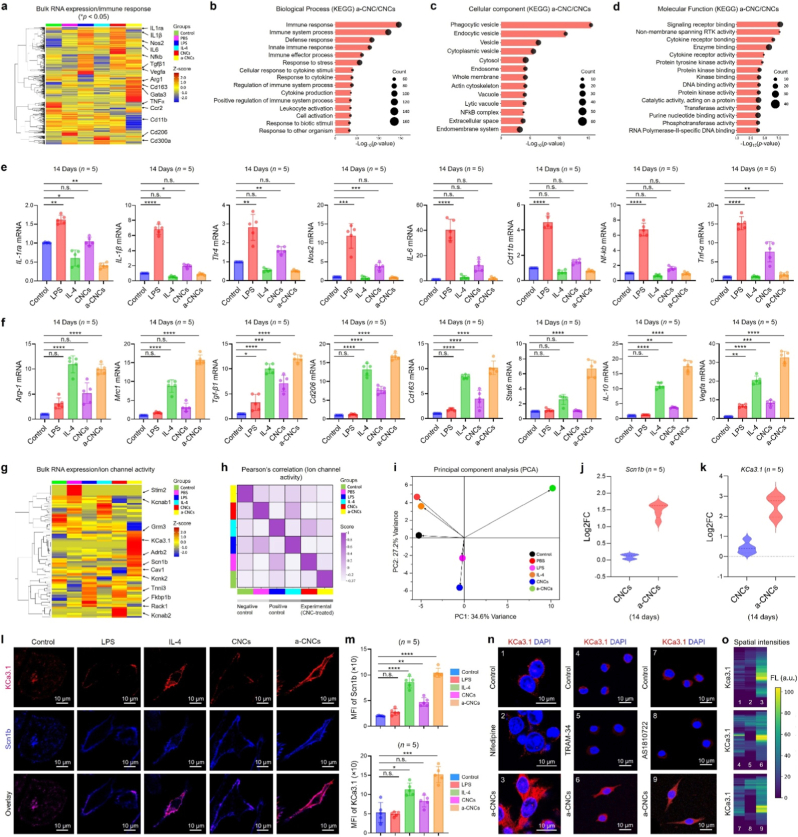
Bulk RNA-Seq analysis of RAW 264.7 cells *in vitro*. **(a)** Unsupervised hierarchical clustering of DEGs (Log2FC, ∗*p* < 0.05) associated with the immune response in RAW 264.7 cells after 14 days across various groups. **(b**–**d)** Representative KEGG enrichment analysis of key DEGs associated with biological process (BP), cellular component (CC), and molecular function (MF) in RAW 264.7 cells. **(e, f)** qRT-PCR validation of canonical M1 and M2 macrophage-specific gene markers expression in various groups at day 14 (*n* = 5 each). **(g)** Unsupervised hierarchical clustering of DEGs (Log2FC, ∗*p* < 0.05) associated with ion channel activity in RAW 264.7 cells after 14 days in various groups. **(h)** Pearson's correlation analysis of key DEGs associated with ion channel activity across groups. **(i)** Principal component analysis (PCA) analysis of variance in various treatment groups at day 14. **(j, k)** Fold change expression of *Scn1b* and *KCa3.1* (=*Kcnn4*) (Log2FC, ∗*p* < 0.05) associated with ion channel activity (*n* = 5 each). **(l, m)** Representative immunostaining results for KCa3.1 (red) and Scn1b (blue) in various groups at day 14. Scale bar: 10 μm (*n* = 5 each). **(n, o)** Immunostaining results with spatial fluoresence intensities for KCa3.1 and Stat6 in RAW 264.7 after ion channel inhibition using nifedipine (5 μM, L-type Ca^2+^ ion channel blocker), TRAM-34 (10 μM, Ca^2+^-activated K^+^ blocker), and AS1810722 (10 μM, Stat6 blocker) at day 14. Scale bar: 10 μm. Data reported as mean ± s.d. of replicated experiments (*n* = 5 each), statistical significance was considered at ∗*p* < 0.05, ∗∗*p* < 0.01, ∗∗∗*p* < 0.001, and ∗∗∗∗*p* < 0.0001 (One-way ANOVA test with Tukey's test *post hoc* analysis).

Inspired from the outstanding *in vivo* biocompatibility and single-cell transcriptomic study, we next studied the effect of pristine CNCs and a-CNCs on *in vitro* macrophage polarization using RAW 264.7 cells via bulk RNA-Seq within the 14-day subacute window and the results are shown in Fig. 4. It has been reported that during initial phase of tissue healing (typically 0 h-3 days, acute stage), M1 macrophages are dominant, while at later stage of healing (>3-5 days, subacute-prohealing stage) M2 phenotypes are dominant as it contributes to anti-inflammatory cytokines activation and extracellular matrix (ECM) remodeling [[26], b), c), a)]. Therefore, we studied the *in vitro* transcriptional changes in RAW 264.7 cells upon CNCs and a-CNC treatment within 14-day subacute window. For bulk RNA-Seq, the RAW 264.7 cells were cultured in the presence of pristine CNCs and a-CNCs (100 μg mL^−1^) for 14 days. LPS and IL-4 (100 ng mL^−1^ each) were used as positive controls for classical M1 and M2 polarization, respectively, while only PBS was used as the negative control.

As shown in Fig. 4(a), the hierarchical clustering of DEGs (Log2FC, ∗*p* < 0.05) associated with immune response showed that RAW 264.7 cells treated with pristine CNCs or a-CNCs clustered closely with PBS-treated group, while a distinct separation was observed when compared with LPS and IL-4-treated groups at day 14, respectively. The key pro-inflammatory (*e*.*g*., *Il-1β*, *Nos2*, *Tnf-α*, *Il-6*, and *Nf-kB*) DEGs remained at a basal level. At the same time, significantly higher expression was observed for key anti-inflammatory genes (e.g., *Arg-1*, *Mrc1*, *Cd206*, *Tgf-β1*, *Vegfa*, and *Cd163*) in the a-CNC-treated group compared with the PBS- and LPS-treated groups. Notably, the expression of various DEGs was similar to that in IL-4-induced cells, suggesting a pronounced shift towards the M2 phenotype. Moreover, *k*-means clustering of immune-related DEGs revealed that RAW 264.7 cells treated with a-CNCs display distinct macrophage polarization genes, which are more closely associated with the IL-4 group than with pristine CNCs or LPS-induced groups (Fig. S7(a)). Notably, both *t-distributed Stochastic Neighbor Embedding* (t-SNE) and *Principal Component Analysis* (PCA) confirmed that a-CNCs-treated RAW 264.7 cells form a large transcriptional cluster enriched with genes related to M2 polarization (*e*.*g*., Arg*1*, *Tgf-β1*, and *Cd163*) and tissue healing pathways (Fig. S7(b and c)), indicating that a-CNC exposure is strongly associated with M2 macrophage polarization. This was also reflected in gene ontology (GO) and KEGG functional enrichment analysis of transcriptome data. As depicted in Fig. S7(d), the KEGG analysis uncovered higher enrichment and gene counts for '*JAK/STAT signaling*,' '*ion channel activity*,' and '*cytokine activity*' when comparing between a-CNCs *vs*. CNCs groups. Furthermore, the *Gene Set Enrichment Analysis* (GSEA) confirmed that a-CNCs drive expression of specific genes that overlap strongly with those induced by IL-4, but not CNCs, indicating that surface activation and involvement of surface functional groups in CNC are essential for immune-interactive functionality (Fig. S7(e)). Besides, the KEGG linked to *Molecular Signature Database* (MSigDB) analysis further suggested *JAK/STAT signaling* (NES: 0.467123, *p* = 0.0001) and *voltage-gated ion channel activity* (NES: 0.727614, *p* = 0.0001) as significant drivers of M2 polarization (Fig. S7(f)), indicating that a-CNC exposure correlates with enhanced ion channel and Stat6 signaling [[27], b), a)]. Next, we performed *Weighted Gene Co-expression Network Analysis* (WGCNA) to identify the gene co-expression network among genes associated with immune response across all samples. As illustrated in Fig. S7(g–i), the WGCNA identified distinct gene modules responsive to a-CNCs, with downstream enrichment for the leukocyte-mediated repair response and up-enrichment for immune response and extracellular matrix remodeling, which are critical features of M2 polarization. Concurrently, the *Pearson Correlation Analysis* (PCA) of the most significant polarization genes showed that a-CNC treatment selectively enhances expression correlation among the top 50 M2-associated genes, confirming activation of a coherent pro-healing phenotype at day 14 (Fig. S7(j–n)).

Patel et al. [3] reported that pristine CNCs (rod/round-shaped particles, –SO_3_H, –COO^-^, and –OH groups) synthesized through acid hydrolysis or ammonium persulfate (APS) oxidation exhibited M2 macrophage polarization at day 1, while mostly showed M1 polarization towards M1 phenotype at day 3. In other studies, CNCs with variable lengths (∼150-720 nm) induced M1 polarization of alveolar macrophages after 24 h by upregulating *Il-4a*, *Ccl4*, *Tnf-α*, and *Il-6*, and by activating NLRP3-mediated inflammasome signaling [6,28]. These reports clearly indicate that CNCs without any surface modification and variable length or size only trigger M1 polarization with pro-inflammatory cytokines production. Consistent with these reports, our results also suggest that pristine CNCs with an average length of ∼140 nm and surface –OH/–SO_3_H groups activated the M1 phenotype, while a notable shift towards M2 phenotype was only observed in the presence of a-CNCs with surface –NH/–NH_2_ groups (positively-charged groups) confirmed that surface functional groups play significant role in macrophage cell polarization *in vitro* [1,29]. To validate this statement and bulk RNA-Seq data, we then performed a qRT-PCR analysis of key M1 and M2 polarization gene markers expression at day 14 after CNCs and a-CNCs treatment. As shown in Fig. 4(e), the key M1-specific gene markers, such as *Il-1ra*, *Il-1β*, *Tlr4*, *Nos2*, *Il-6*, *Cd11b*, *Nf-kB*, and *Tnf-α,* were significantly down-regulated in the a-CNCs group, while a slight up-regulation was found in the pristine CNCs group, suggesting that pristine CNCs consistently promoted M1 polarization of RAW 264.7 cells at day 14. Similarly, a remarkable enhancement of gene markers expression, such as Arg*1* (>5.0 fold), *Mrc1* (>10.0 fold), *Tgf-b1* (>10.0 fold), *CD206* (>15.0 fold), *CD163* (>5.0 fold), *Stat6* (>4.0 fold), *Il-10* (>15.0 fold), and *Vegfa* (>20.0 fold) were found significantly upregulated in a-CNCs-treated group than other groups at day 14 (Fig. 4(f)). This confirms that surface-activated CNCs (–NH/–NH_2_) intrinsically promote M2-like macrophage polarization without eliciting inflammatory signatures.

Ion channel activation is another critical phenomenon for macrophage polarization and disease progression [[30], a), b)]. Since preliminary transcriptomic data suggested the involvement of ion-channel-related pathways, we then investigated the various ion channel activity-related DEGs associated with CNCs and a-CNCs treatment at day 14. Fig. 4(g) shows the unsupervised clustering of DEGs associated with ion channel activity in RAW 264.7 cells. Interestingly, the a-CNCs induced a distinct expression pattern characterized by the significant upregulation of Ca^2+^/K^+^ ion channels (*e*.*g*., *Kcnn4*/*KCa3.1*, *Trpv2*, and *Trpv4*), followed by various Na^+^/Cl^−^ voltage-gated channel regulators (*e*.*g*., *Scn1b*, *Scn10a*, *Clcn3*, and *Clcn7*), which are mainly expressed during M2 macrophage polarization [[31], b), c), a)]. Moreover, the Pearson correlation analysis revealed strong co-regulation among these genes only in the a-CNC group. This pattern was completely different in the PBS-, LPS-, IL-4-, or CNC-treated groups (Fig. 4(h)). Furthermore, the PCA plot showed greater variance in ion channel-related genes in the a-CNC group than in the other groups, highlighting the unique cell membrane excitability and signaling facilitated by a-CNCs (Fig. 4(i)). The fold change expression of *Scn1b* and *KCa3.1* was found significantly higher in a-CNC-treated groups than CNCs, as indicated in the violin plots (Fig. 4(j and k)). A STRING protein-protein interaction study of all identified ion channel-responsive genes is shown in Fig. S8. Studies have shown that *KCa3.1*, a product of *Kcnn4* of the K^+^ ion channel family, regulates macrophage nucleation and differentiation via activating IL-4 type 1 receptor [[32], a), b)]. In contrast, *Scn1b* directly activates the JAK/STAT signaling and promotes the M2 macrophage polarization [33]. To validate this, we examined KCa3.1 and Scn1b expression levels by immunostaining. As depicted in Fig. 4(l), a remarkable enhancement of cytoplasmic and membrane-bound fluorescence was spotted in the a-CNCs group, which was comparable with the IL-4 group. Besides, the pristine CNCs groups showed negligible fluorescence for both KCa3.1 and Scn1b. The statistical analysis further confirmed a substantial increase (∗∗∗∗*p* < 0.0001) in mean fluorescence intensity of KCa3.1^+^ and Scn1b^+^ cells (Fig. 4(m)), suggesting that ion channel activation is strongly associated with M2 polarization after a-CNCs exposure. Similarly, this pattern was more prominent when we used a L-type ion channel blocker (Nifedipine/5 μM) and Ca^2+^-activated K^+^ ion channel blocker (TRAM-34/10 μM) to study the expression of *KCa3.1*. As shown in Fig. 4(n), ion channel blockage significantly reduced the expression of *KCa3.1*, which was found to overexpress in a-CNC-treated group at day 14 subacute window, underscores that activation of Ca^2+^ ion channel is primarily associated with a-CNC-mediated M2 macrophage polarization in RAW 264.7 cells. Furthermore, *Stat6* inhibition using AS1810722 (10 μM) also revealed a downregulation of *Stat6* expression in RAW 264.7 cells when compared with a-CNCs group, concurrently suggesting that a-CNCs treatment positively activates both Ca^2+^-ion channels and *Stat6* gene during macrophage polarization. The spatial intensity analysis for *KCa3.1* upon pharmacological inhibition is shown in Fig. 4(o). In parallel, the pharmacological inhibition displayed significantly low expression of *KCa3.1* (∼0.5-0.8 fold) and *Stat6* (∼0.5-0.6 fold) gene markers in RAW 264.7 cells at day 14, while a-CNC-treated groups maintained a significantly (∗∗∗∗*p* < 0.0001) higher expression of *KCa3.1* (>10.0 fold) and *Stat6* (>8.0 fold) genes at day 14 (Fig. S9). Strikingly, the live cell Ca^2+^ imaging study revealed a clear evidence of intracellular Ca^2+^ gating from control to a-CNCs, with the a-CNCs group significantly higher fluorescence intensity and highest density of Ca^2+^-active cells (Fig. S10(a)). Correspondingly, spatial Ca^2+^ intensity maps demonstrated a broader distribution and highest single-cell fluorescence signals in the a-CNCs groups (Fig. S10(b)). The a-CNCs typically binds and/or internalized in RAW 264.7 cells, resulting in Ca^2+^ influx, which selectively activates KCa3.1 channels and causes membrane hyperpolarization, followed by enhanced Ca^2+^ signaling. This enhancement in Ca^2+^ regulate downstream transcription factor Stat6, a key transcription factor of M2 macrophage polarization [34].

### 2.5 *In vitro* validation of macrophage polarization

**Fig. 5:**
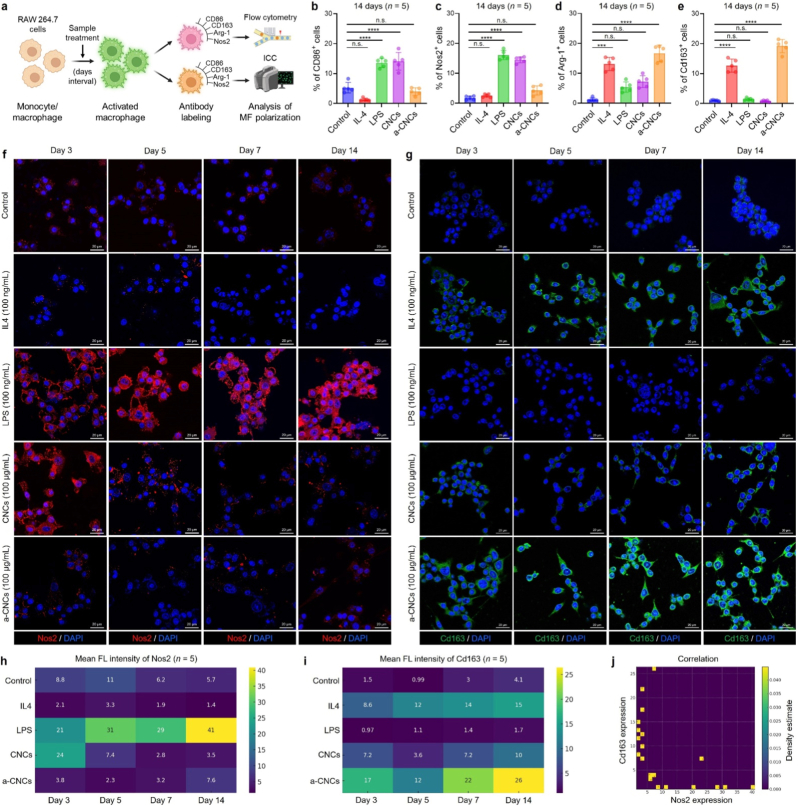
*In vitro* validation of macrophage polarization using pristine CNCs and a-CNCs. **(a)** Schematic outline of the macrophage polarization study. **(b**–**e)** Representative flow cytometry analysis of the RAW 264.7 cells showing the expression of M1 (Cd86 and Nos2) and M2 (Arg-1 and Cd163) markers in various groups at day 14 (*n* = 5 each). **(f, g)** Immunostaining images of RAW 264.7 cells showing the expression of Nos2 and Cd163 protein marker at the indicated time points. Scale bar: 20 μm. **(h, i)** Representative fluorescence intensities of the Nos2 and Cd163 at the indicated time points (*n* = 5 each). **(j)** 2D joint density plot showing the correlation between Nos2 and Cd163 expression at day 14. Data reported as mean ± s.d. of replicated experiments (*n* = 5 each), statistical significance was considered at ∗*p* < 0.05, ∗∗*p* < 0.01, ∗∗∗*p* < 0.001, and ∗∗∗∗*p* < 0.0001 (One-way ANOVA test with Tukey's test *post hoc* analysis).

The *in vitro* transcriptomic data were validated by flow cytometry, immunostaining, and cytokine profiling in RAW 264.7 cells cultured for up to 14 days with CNCs or a-CNCs. Fig. 5(a) shows a schematic illustration of the *in vitro* validation study of macrophage polarization. Flow cytometry analysis at day 14 revealed a marked shift in macrophage polarization (Fig. 5(b and c)). In particular, the a-CNCs treatment significantly downregulated the abundance of Cd86^+^ and Nos2^+^ populations compared to pristine CNCs. At the same time, a marked increase in Arg-1^+^ and Cd163^+^ cells were observed at day 14 (Fig. 5(d and e)), suggesting a dramatic shift towards an M2 phenotype. This was further correlated with the morphological analysis of RAW 264.7 cells over 14 days. Fig. S11 shows the dynamic morphological and functional polarization behavior of RAW 264.7 cells following long-term exposure to CNCs and a-CNCs. Bright-field images reveal that PBS-treated and CNC-treated cells retain a round, undifferentiated morphology, while a-CNC–treated macrophages progressively adopt an elongated spindle-like shape by day 14, a signature of a well-established phenotype of M2-polarized macrophages similar to the IL-4-treated group (Fig. S11(a)). In contrast, LPS-treated cells maintain a flattened and poached-egg-like morphology characteristic of M1 activation. Statistical analysis for % of elongated cells confirms this morphological shift, with a-CNCs inducing a significantly (∗∗∗∗*p* < 0.0001) higher % of elongated M2-like macrophages up to 14 days (Fig. S11(b–e)). CNCs displayed only minimal elongation, reinforcing their typical M1 polarization, consistent with previous reports [3]. More interestingly, the cell aspect ratio measurements show that the a-CNC-treated group exhibits increased cellular area over time (Fig. S11(f–i)), resembling IL-4-induced hypertrophy associated with enhanced phagocytosis and tissue-repair functionality, whereas CNCs do not stimulate notable morphological enlargement.

Time-dependent (3, 5, 7, and 14 days) immunostaining of RAW 264.7 cells showed a gradual decrease in Nos2 fluorescence from day 3 to day 14 in the a-CNC-treated group, which was comparable to that observed with IL-4 treatment (Fig. 5(f)). Interestingly, Cd163 fluorescence was significantly increased when we moved from the control to the a-CNCs group over a time frame of 3-14 days (Fig. 5(g)). The control group exhibited negligible fluorescence for both Nos2 and Cd163, suggesting their undifferentiated (=unpolarized) state. The statistical data from immunostaining indicated a significantly (∗∗∗∗*p* < 0.0001) higher fluorescence intensity for Cd163 than for Nos2 in the a-CNC-treated group (Fig. 5(h and i)). The higher-magnification single-cell fluorescence images are shown in Fig. S12 and Fig. S13. Moreover, the 2D joint density plot further suggests an overall inverse relationship between Nos2 and Cd163 expression, with mostly clustering at low Nos2/high Cd163 conditions (Fig. 5(j)). Taken together, consistent with scRNA-Seq and bulk RNA-Seq data, the *in vitro* findings demonstrate that surface-activated a-CNCs (–NH/–NH_2_) actively promote morphological reprogramming of RAW 264.7 cells toward an M2-like, pro-healing phenotype**,** validating their intrinsic immunomodulatory potential. A comparative study on various surface-functionalized nanocellulose and its *in vitro*/*in vivo* effect on macrophage fate is shown in Table S1.

## 3 Conclusion and outlook

**Scheme 2:**
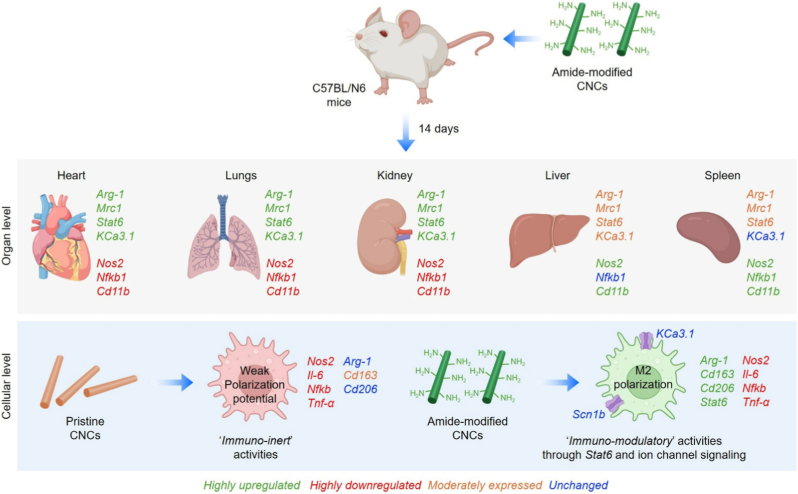
Schematic illustration showing the effects of amide-modified CNC on macrophage fate determination *in vivo*.

In summary, our findings suggest that amide-functionalized a-CNCs is an immunocompatible nanomaterial capable of orchestrating tissue-resident macrophage identity across diverse organ niches. Surface amide decoration transforms CNCs from an ‘*immuno-neutral*’ to an ‘*immuno-instructive*’ material by activating ion channels in macrophages. Through an integrated multi-omics strategy, combining scRNA-Seq, bulk RNA-Seq, histology, and functional assays, we demonstrate that a-CNCs maintain systemic immune homeostasis while eliciting a robust pro-healing macrophage phenotype driven by *Stat6* and voltage-gated ion channel (*KCa3.1* and *Scn1b*) activation, and suppression of *Nfkb*-dependent inflammatory signaling. Importantly, a-CNCs display macrophage developmental trajectories without hindering niche architecture, whereas pristine CNCs induce mild and organ-specific M1 macrophage polarization, particularly in the spleen. These findings underscore that '*nano-immune*' interaction can be tailored through CNCs' surface chemistry, redefining its therapeutic potential beyond its conventional roles in structural reinforcement or drug delivery. An overview of the major outcomes of this study is schematically illustrated in Scheme 2.

Although a-CNCs induced a macrophage phenotype, genetically and transcriptionally aligned with pro-healing M2 programs, direct tissue-level functional outcomes (*e*.*g*., regeneration, fibrosis resolution, or repair kinetics) were not assessed in this study and remain important directions for future investigation. Generally, various pathological situations (*e*.*g*., infection, trauma or injury) alter inflammatory cytokines, induce hypoxia or metabolic stress, which may significantly alter immunomodulatory pathways, including ion channel activity and Stat6 activation. In particular, a-CNCs-incorporated bioactive hydrogels would serve as a promising platform for immunomodulation-assisted tissue healing and regeneration. Moreover, integrating a-CNCs with stimuli-responsive hydrogels, controllable degradation profiles, or bioactive co-factors may enable temporal control over macrophage transitions during tissue repair. At a fundamental level, the transcriptional and pharmacological immune reprogramming observed here provides deeper insight into how a-CNCs ‘*nanoscale*’ chemical cues regulate ‘*macroscale*’ immunomodulation, mechanotransduction, and cell fate decisions.

## 4 Experimental section

### 4.1 Synthesis and amide functionalization of nanocellulose

Bulk cellulose from cotton pulp was obtained from the Institute of Forest Science at Kangwon National University. The rod-shaped cellulose nanocrystals (CNCs; yield: ∼42.9%) were synthesized via acid hydrolysis, as reported previously [35]. The 2,2,6,6-tetramethylpiperidine-1-oxyl (TEMPO)-mediated oxidation of CNCs (t-CNCs) was carried out as reported in the literature [10]. The yield of t-CNCs was around ∼36.7%. The amine-functionalized carbon dots (N-CDs; yield: ∼77.2%) were synthesized as reported in our previous study [10]. The CNCs modification with N-CD was carried out using 1-(ethyl-3-(3-dimethylaminopropyl)-carbodiimide/N-hydroxyl succinimide (EDC/NHS) chemistry. In a typical synthesis, 10% t-CNCs (*w*/*v*) were taken in a 500 mL beaker and stirred for 30 min, followed by the addition of 8.3 g EDC (Sigma-Aldrich) and 32 g NHS (Sigma-Aldrich) dropwise for 2 h to charge the carboxylic groups. After 2 h, 100 mL of N-CDs (2 mg mL^−1^) was added to the solution and stirred vigorously overnight at room temperature. Next, the resulting solution was centrifuged at 13,000 rpm, washed three times with D.I. water to remove unbound CDs, and dialyzed (M.W. Cut-off: 12-14 kDa, Millipore) for at least 3 days. The residual paste was freeze-dried to obtain CDs-modified CNCs (a-CNCs; yield: ∼40.3%). The product was stored at 4 °C until further use.

### 4.2 General characterizations

The surface charge of the as-synthesized CNCs, t-CNCs, and a-CNCs was examined using zeta potential (Zetasizer, Malvern PANalytical). The carboxylic moieties in the samples were verified using toluidine blue-O staining (TBO; Sigma-Aldrich), as reported in our previous study [10]. The morphologies of the CNCs, t-CNCs, and a-CNCs were investigated using a transmission electron microscope (TEM; JEOL). The structural properties of the samples were analyzed using X-ray diffraction (XRD; X'Pert Pro MPD) spectroscopy equipped with a CuK α monochromatic gun ($\lambda =1.54\;\dot{A}$). The chemical moieties and surface functional groups of the samples were analyzed using a Fourier transform infrared (FT-IR; Frontier, PerkinElmer) spectroscopy and X-ray photoelectron spectroscopy (XPS; Thermo-Fischer Scientific). A detailed characterization of N-CDs is reported in our previous study [10].

### 4.3 Cell viability assays

The murine monocyte/macrophage RAW 264.7 cells were obtained from the Korean Collection for Type Cultures (KCTC, AC28116). The cells were found positive for Cd11b, Cd14, Cd36, iNOS (=Nos2), Vegfr2, and Hif-2 α with stable expression and negative for Arg-1 and Tgf-β 1. The *in vitro* cytotoxicity of RAW 264.7 cells was assessed using a WST-8 assay after 24 h of incubation. We used the CNCs (first product) and a-CNCs (end product) for all the biological experiments. We excluded the t-CNCs (intermediate product) from the macrophage polarization study unless stated otherwise. For the WST-8 assay, RAW 264.7 cells (∼1.5 × 10^4^) were cultured in DMEM (Welgene) media supplemented with 10% FBS (Thermo-Fischer Scientific) overnight. After that, various concentrations of CNCs and a-CNCs (0-500 μ g mL^−1^) were treated and incubated for 24 h. After that, the cells were incubated with WST-8 dye, and RAW 264.7 cell viability was assessed by measuring optical density (O.D.) at 450 nm using a spectrophotometer (Infinite M Nano, TECAN).

### 4.4 *In-vivo* macrophage polarization study

#### 4.4.1 Animal care and maintenance

C57BL/N6 male mice were obtained from Orient Bio Inc., Gyeonggi-do, Republic of Korea. 8-12-week-old mice (body weight: 25-30 g) were used for the macrophage polarization study. The mice were randomly divided into three groups: (1) Control groups (saline, *n* = 10), (2) CNCs group (100 μ g mL^−1^, *n* = 10), and (3) a-CNCs group (100 μ g mL^−1^, *n* = 10). The mice euthanasia was carried out using an isoflurane gas chamber equipped with an antibacterial filter system for controlled inhalation. All the animal experiments were approved by the Institutional Review Board (IRB) of the Institute of Animal Care and Use Committee (IACUC) of Korea National University of Transportation Hospital (Permission No. KNUT-2024-A3E). The samples were UV sterilized before application.

#### 4.4.2 Sample treatment and biodistribution study

Prior to sample treatment, the mice were fed sufficient food and water. Next, the 100 μL of CNCs (100 μ g mL^−1^/10 g bw) and a-CNCs (100 μ g mL^−1^/10 g bw) were injected (*i.v.*) through the tail. At around 12 h post-injection, the major organs (heart, lung, kidney, spleen, and liver) were removed from the body, and the biodistribution was recorded using an IVIS system (PerkinElmer) with excitation/emission at 408/620 nm, respectively. The mean fluorescence intensity (MFI) was analyzed by drawing a region of interest (ROI) for each organ.

#### 4.4.3 Tissue-specific macrophage polarization study

The tissue-specific macrophage polarization potential of the CNCs and a-CNCs was studied after macrophage depletion following clodronate injection for 48 h [36]. Briefly, after isoflurane anesthesia, 0.2 mL of clodronate liposome (Creative Biolabs) was injected into the retro-orbital sinus of each mouse. The dose was repeated after 24 h. The macrophage depletion was validated by monitoring the expression of F4/80^+^ resident macrophages in the spleen via flow cytometry (BD Accuri™ C6) and immunocytochemistry (DMi8, Leica Microsystems) against MHCII APC. We observed successful macrophage depletion (∼98.7%) after 48 h (2 doses at 24 h intervals) by verifying a reduction in F4/80^+^ cell population in the spleen. We also validated the serum TNF-α, IL-1 β, and IL-6 expression after clodronate injection. Immediately after 48 h of macrophage depletion, each mouse received a dose of 100 μ g mL^−1^ of CNCs and a-CNCs for up to 14 days. After 14 days, the mice were sacrificed, and major organs were harvested for a macrophage polarization study.

#### 4.4.4 Histology, biochemistry, and immunofluorescence study

After 14 days of CNCs and a-CNCs administration, *in vivo* macrophage polarization was studied by extracting the major organs (heart, lung, kidney, spleen, and liver) and staining them with multicolor immunofluorescence. The organ samples were washed with saline, then fixed with paraformaldehyde (PFA; Sigma-Aldrich) for 24 h. After fixation, the samples were paraffin-embedded and sectioned with a cryo-ultramicrotome (CM 3050S, Leica) to produce 5 μm-thick sections. For histopathology, the organ sections were stained with hematoxylin and eosin (Sigma-Aldrich) to assess *in vivo* biosafety. For the immunofluorescence study, the frozen section was fixed with acetone (Sigma-Aldrich) for 10 min, followed by permeabilization with 0.1% Triton-X 100 (Sigma-Aldrich) for 15 min. After that, the tissue sections were blocked with 1% BSA (Sigma-Aldrich) for 1 h at room temperature. Following 3-5 times washing with 1 × PBS, the tissue sections were incubated with primary antibodies against Nos2 and Cd163 (Santa Cruz Biotechnology) overnight at 4 °C. After that, the samples were washed 3-5 times with 1 × PBS and incubated with appropriate secondary antibodies (Abcam and Santa Cruz Biotechnology) for 2-3 h at room temperature. All the samples were stained with DAPI (Sigma-Aldrich) for 10 min at room temperature and finally mounted with an Antifade mounting media (Prolong™, Thermo Scientific). Expression of various markers was visualized using an inverted fluorescence microscope (DMi8, Leica) with appropriate filter sets. Detailed information on the antibodies used for immunofluorescence is provided in Table S2. A routine blood biochemistry analysis was also performed using fresh blood collected at day 14.

#### 4.4.5 Macrophage isolation and sorting for single-cell RNA sequencing

The tissue-resident macrophages were extracted from the corresponding tissues after 14 days of sample (CNCs and a-CNCs) treatment. The fresh organs were harvested from the mouse and immediately stored using MACS tissue storage solution (Miltenyi Biotech) at 4 °C. The macrophages were then isolated and purified from the respective tissue as reported elsewhere with slight modifications. The isolation protocols are as follows.

The heart- and kidney-resident macrophages were isolated from the myocardial specimen using the enzymatic digestion technique, as previously reported [37]. Briefly, the minced myocardial tissue (from whole heart) was taken in a sterile 50 mL falcon tube (BD Biosciences) and treated with 3 mL of an enzymatic cocktail containing Collagenase-I (450 U mL^−1^, STEMCELL Technologies), DNase-1 (60 U mL^−1^, Takara Bio), and Hyaluronidase (60 U mL^−1^, Sigma-Aldrich) in DMEM (Welgene) media. The mixture was vortexed for 30 s and incubated in a shaking water bath at 37 °C for 1 h. After that, the tubes were placed on ice for a few min, and the digested tissue solution (30 mL/tube) was sieved through a 40 μ m cell strainer (Corning®), followed by the addition of enzyme deactivating (ED) buffer (16 mL/tube). The solution was mixed thoroughly and again sieved through a 40 μ m strainer, keeping 15 mL of cell solution per tube. The solution was centrifuged at 400×*g* for 6 min at 4 °C. Afterward, the supernatant was discarded, and the pellet was resuspended in 1 mL of ACK lysis buffer (Thermo Scientific) and gently pipetted. The mixture was incubated for 5 min at room temperature. This will enhance the RBC lysis in the ACK medium. After 5 min, 9 mL of fresh DMEM media was added to the tube (containing 1 mL of ACK buffer with cells) and mixed by rotating the Falcon tube upside down at least 3-4 times. The resulting solution was then passed through a 40 μ m filter, and the single-cell suspension was collected (∼15 mL/extraction). The solution was recentrifuged at 400×*g* for 6 min, and the pellet was resuspended in 1 × PBS. The cells were finally collected and resuspended in a staining buffer containing 0.5% BSA (Sigma-Aldrich) in 1 × Ca^2+^/Mg^2+^-free Hank's balanced salt solution (HBSS, Welgene) for FACS antibody staining and later sample preparation.

The lung and alveolar macrophages were purified using an enzymatic digestion technique, as reported elsewhere [38]. Briefly, the mice were anesthetized in an isoflurane vapor chamber, followed by intracardial perfusion with 1 × Ca^2+^/Mg^2+^-free HBSS to remove blood cells. Next, the blood-free whole lung was transferred into a Petri dish containing 15 mL of RPMI-1640 media (Welgene) and minced carefully with sterile forceps. After that, the minced lung tissue was taken into a sterile falcon tube and digested with RPMI-1640 media (15 mL) containing Collagenase-IV (0.5 mg mL^−1^, STEMCELL Technologies) for 10 min, DNase-1 (0.02 mg mL^−1^, Takara Bio) for another 20 min, and again digestion with a mixture of Collagenase-IV + DNase-1 (5 mL in RPMI media) for 10 min at 37 °C under gentle agitation. After final digestion, the mixture was sieved through a 100 μ m cell strainer (BD Biosciences) to collect the single-cell suspension. After that, the collected cells were washed thrice with 1 × PBS, centrifuged (400×*g* for 6 min), and resuspended in 1 × HBSS for FACS analysis.

The spleen-resident macrophages and spleenocytes were isolated using tissue grinding and RBC lysis. Briefly, after isoflurane anesthesia, the spleen was carefully dissected from the mouse and rinsed with 1 × HBSS, followed by mechanical grinding in a sterile mortar pestle with RPMI-1640 media supplemented with 5% FBS (Gibco-BRL). After that, the dissociated tissue mixture was poured into a 50 mL Falcon tube using a 100 μ m cell strainer (BD Biosciences). The resulting cell suspension was centrifuged at 400×*g* for 6 min, and the supernatant was discarded. The pellet was then rinsed with ice-cold 1 × PBS (Welgene), followed by incubation in 1 × RBC lysis buffer (Roche, Sigma-Aldrich) on ice for 3 min to remove RBC contamination. Finally, the resulting solution was centrifuged at 400×*g* for 6 min, washed twice with 1 × HBSS, and stained for FACS analysis.

For liver-resident macrophages, we employed a two-step perfusion digestion strategy for hepatic cells and a density gradient centrifugation (Optiprep™) technique, as described previously. Briefly, the dissected liver (whole) was subjected to a laparotomy in order to expose the liver lobules (LL), portal veins (PV), and inferior vena cava (IVC). In the first step, the liver was perfused with warm (37 °C) 1 × HBSS supplemented with 1.9% EGTA (Sigma-Aldrich) until a transparent liquid began to flow. In the second step, perfusion was performed to remove all RBC traces from the liver using 1 × HBSS supplemented with 1% FBS (Gibco-BRL) and a digestive enzyme mixture containing Collagenase-IV (0.5 mg mL^−1^, STEMCELL Technologies) and calcium chloride (5.6%, Sigma-Aldrich). This allowed the liver tissue to become nonelastic, and at this point, the peristaltic pumping (SciTech) through PV to IVC must be stopped. After that, the liver was gently detached from the mouse and placed on a sterile Petri dish containing fresh DMEM with 0.5% BSA. Following superfine mincing, the tissue aggregates were passed through a 100 μ m cell strainer (BD Biosciences) to obtain single-cell suspensions. At this point, the parenchymal liver cell pellet was discarded following low-speed centrifugation (50×*g*) for 2 min at 4 °C. Next, the supernatant was collected and recentrifuged at 500×*g* for 8 min at 4 °C to collect the nonparenchymal liver cells, which mostly contain macrophage subpopulations. To purify macrophage cells, we next performed a density gradient centrifugation using Optiprep™ (Axis-Shield; 0%, 11.2%, 17.6%, and 24%) by centrifuging at 1400×*g* at room temperature for 20 min. After that, the fraction between 11.2 and 17.6% was gently collected using a transfer pipette (Eppendorf), washed 3 times with 1 × HBSS buffer, and resuspended in HBSS for FACS analysis.

For cell sorting (FACS) analysis, the single-cell populations were incubated with a mouse Fc blocker (AAT Bioquest) for 20 min on ice, followed by staining with anti-CD45 (1:200, BD Biosciences). Its corresponding Alexa Fluor-488-conjugated secondary antibodies (1:200, BD Biosciences) in ice-cold BSA (1% in PBS) for 30 min. After the desired staining, the cells were washed twice with PBS, sieved through a 40 μm cell strainer (Falcon, BD Biosciences), and analyzed using a BD FACS Aria-III (BD Biosciences) equipped with a 100 μm capture nozzle and appropriate excitation lasers. The raw data were analyzed with *FlowJo*™ software (v10.7, BD Biosciences). The list of antibodies used in FACS analysis is given in Table S2.

#### 4.4.6 Library preparation and single-cell RNA sequencing

The Cd45^+^ single cells from each organ in different groups were directly used for library preparation and single-cell RNA (scRNA-Seq) analysis. Briefly, the cells were counted (∼1.2 × 10^4^ ml^−1^), suspended in 0.04% BSA (prepared in 1 × PBS), and processed with a Chromium single-cell 3*′* library kit and gel bead kit (10X Genomics) according to the manufacturer's guidelines. After desirable lysis and treatment, the single-cell gene libraries were sequenced on the NovaSeq 6000 (Illumina) sequencer at a read depth of ∼60,000 reads per cell for total expression libraries and 12,000 reads per cell for TCR libraries.

The raw Illumina sequencing data were exported as basecall (∗.bcl) files and converted to fastqs files using *Cell Ranger* (v3.0.1, 10X Genomics) with standard algorithms, followed by statistical analysis. Next, the FASTQ files were then processed with the *Seurat* (v5.1) R package for clustering and visualization, and compared using a mouse mm39 (*Mus musculus*, GCF_000001635.27) reference genome with a threshold of >5000 variables. The obtained data were plotted using *Uniform Manifold Approximation and Projection* (UMAP) clustering of the top 3 principal components, i.e., TLF^+^, MHC-II^+^, and Ccr2^+^ populations, with k-nearest neighbors (KNN) analysis to identify differentially expressed genes associated with macrophage polarization in various organs upon CNCs and a-CNCs treatment in *vivo*. The defined clusters were then screened for the macrophage polarization markers, and their gene expression was studied. We also performed the cell doublet detection and removal analysis to exclude the signature genes that were expressed by the Monocyte-T cell doublets. The expression of major macrophage polarization (M1 and M2) genes was plotted using the *Heatmapper* R package (RStudio). The Gene Set Enrichment Analysis (GSEA v4.0.2) was used to identify significant up- and down-regulated pathways across groups.

### 4.5 *In-vitro* validation of macrophage polarization

#### 4.5.1 In-vitro morphological investigations

The RAW 264.7 cells (∼1.5 × 10^5^/100 μL/96-well) were incubated with 1 × PBS (Welgene Inc.), LPS (100 ng mL^−1^, Invitrogen), IL-4 (100 ng mL^−1^, GenScript), and CNCs (bulk CNCs and a-CNCs, 100 μg mL^−1^ each) up to 7 days. The untreated cells were considered the negative control, while the PBS-, LPS-, and IL-4-treated groups were considered the positive controls. All the treatments were performed in replicates (*n* = 5). At various time points (e.g., day 3, day 5, day 7, and day 17), the RAW cells were washed with 1 × PBS, fixed with 1% PFA, and photographed using an inverted optical microscope (Zeiss, Germany) to assess morphological changes. At least five (*n* = 5) independent images per group were collected for semi-quantitative analysis.

#### 4.5.2 In-vitro M1/M2 macrophage polarization study

For this, RAW 264.7 cells (∼2.5 × 10^4^/1 mL/24-well) were incubated with various formulations for up to 7 days. At various time points (*e*.*g*., day 3, day 5, day 7, and day 14), the cells were washed with PBS, fixed with 3.7 % PFA for 15 min at RT. After that, the cells were permeabilized with 100% ice-cold methanol (Sigma-Aldrich) for 10 min at 4 °C, followed by blocking with F_c_ receptor blocker (1:100 in 1% BSA, AAT Bioquest) for 30 min at RT. Following that, the cells were incubated with primary antibodies against anti-iNOS (1:500, SCBT) and anti-Cd163 (1:500, SCBT) for 1 h at RT. After that, the cells were washed with PBS thrice and incubated with AF-594 (1:250, SCBT) and AF-488 (1:250, SCBT) conjugated secondary antibodies for 1 h at RT. Finally, the cells were counterstained with DAPI (Sigma-Aldrich) and mounted with Prolong™ Gold Antifade mounting media (Thermo Scientific). The images were captured using an LSM880 with Airyscan (Zeiss, Germany) equipped with a diode laser (405 nm, 30 mW), a MAr laser (488 nm, 25 mW), and a HeNe laser (594 nm, 25 mW), respectively. The images were acquired with ZEN (v3.2) at 20 × and 40 × magnification. At least three (*n* = 5) independent images were taken from each group for FL intensity measurements. The list of antibodies used in FACS and ICC analysis is given in Supplementary Table 1.

#### 4.5.3 RNA isolation and bulk RNA sequencing

At day 14, the total RNA was extracted from various groups (∼4 × 10^4^ cells/mL/24-well) using TRIzol® (Sigma-Aldrich) reagent according to the manufacturer's guidelines. RNA quality was assessed using a 2100 Bioanalyzer (TapeStation, Agilent), and RNA integrity number (RIN) > 9.0 was used for the 3′ UTR of bulk RNA sequencing analysis. The library construction and QuantSeq 3′ mRNA sequencing were performed using a NexSeq-550 sequencer (Illumina). Next, the FastQC files were aligned with a reference genome (GRCm39, *Mus musculus*, NCBI) to generate a gene *unique molecular identifier* (UMI) matrix. The data were processed using *Excel-based differential expression* gene *analysis* (ExDEGA, v5.2.1, ebiogen Corp.). The differentially expressed genes (DEGs) in the control *vs*. treatment groups were represented as fold change (Log2FC; ∗*p* < 0.05, *t*-test), and normalization was performed using the TMM + CPM method (edgeR), normalized to RC+1.

#### 4.5.4 Unsupervised and k-means hierarchical clustering

Unsupervised and k-means clustering of DEGs (Log2FC, ∗*p* < 0.05, *t*-test) involved in immune response, macrophage polarization, and ion channel activity was performed using the web-based iDEP (v2.0, South Dakota State University). The heatmaps and clustering analysis were performed using standard procedures with *ClustVis* (v2.0). Additionally, the gene ontology (GO) and the involvement of DEGs in various clusters, along with their possible pathways, were visualized using the *Kyoto Encyclopedia of Genes and Genomes* (KEGG) and *DAVID* Functional Annotation Bioinformatics (NIH) tools.

#### 4.5.5 Gene set enrichment analysis (GSEA) for signature genes

We used *GSEA* (v4.3.3, UC San Diego, USA), *GSEA* linked to *the Molecular Signature Database (MSigDB),* and *g:Profiler* (Elixir Infrastructure) to perform GO and KEGG enrichment analyses for signature genes involved in immune response and the macrophage M1/M2 polarization study. Functional enrichment was performed using all available gene sets, with a threshold of ∗*p* < 0.05 (Bonferroni's correction), followed by GO analysis (BP, CC, and MF), biological pathways (KEGG and Reactome), and regulatory databases (miRTarBase).

#### 4.5.6 Weighted gene co-expression network analysis (WGCNA)

Highly correlated gene clusters (modules) across all samples were used to identify hub genes using *Weighted Gene Co-Expression Network Analysis* (WGCNA) in R. 2D principal component analysis (PCA) was performed to visualize DEG variance across samples.

#### 4.5.7 Quantitative real-time PCR (qRT-PCR) analysis

The signature genes involved in M1/M2 macrophage polarization *in vitro*, identified from bulk RNA sequencing data, were validated using qRT-PCR analysis. Genes associated with M1/M2 polarization includes *Il-1ra*, *Il-1β*, *Nos2* (=*iNOS*), *Tlr4*, *Il-6*, *Cd11b*, *Nfkb*, *Tnf*-*a*, Arg*1*, *Mrc-1*, *Tgf-β1*, *Cd206, Cd163*, *Stat6*, *Il-10*, and *Vegfa*, while the ion channel responsive genes include *KCa3.1*, respectively. For indirect validation of *in vitro* macrophage polarization, ICC and morphometric analysis was also conducted. The qRT-PCR analysis was carried out using standard procedures as reported in our previous study [39]. All the primers were designed using *Primer3 Plus* and procured from Bioneer® Corporation, South Korea. A list of gene primers used for qRT-PCR analysis is given in Table S3.

#### 4.5.8 Ion channel and Stat6 inhibition study

For this, the RAW 264.7 cells (∼1.5 × 10^5^/mL/6-well) cultured with a-CNCs (100 μg mL^−1^) and ion channel inhibitors, such as Nifedipine (50 μM, Sigma-Aldrich), TRAM-34 (100 μM, MedChemExpress), and AS1810722 (100 μM, MedChemExpress) for 14 days. The control group received PBS only. At day 14, the cells were stained with primary antibodies against KCa3.1 and Scn1b (Thermo Scientific). The images were captured using an inverted fluorescence microscope (Zeiss) and processed with ImageJ software.

#### 4.5.9 Live cell Ca^2+^ imaging

To visualize the change in intracellular Ca^2+^ influx, RAW 264.7 (∼1.5 × 10^5^/mL/24-well) cells cultured with a-CNCs for 14 days was incubated with Fluo-4 (5 μM, Thermo-Fischer Scientific) for 60 min at 37 °C. After that, the live cell imaging was performed using an inverted confocal microscope (LSM880, Zeiss) and spatial Ca^2+^ intensity from each cell was quantified. The data was represented as pixel-based intensity map.

### 4.6 Software and data analysis

The raw imaging data (ICC and histology) were processed with *LAS-X* (Leica), *ZEN* (v2012, Zeiss), and/or *ImageJ* (v1.8.0, NIH). Cell sorting analysis was performed in FlowJo™ (v10.0). *OriginPro (v9.0, OriginLab*) and *GraphPad Prism* (v10.6.1) were used for graph preparation. The DEG expression and comparison analysis were performed using *ExDEGA* (v5.2.1, ebiogen). The qRT-PCR data analysis was conducted in *CFX Maestro v1.1* (Bio-Rad). All figures were prepared using *Microsoft PowerPoint* (v2016, Microsoft Corp.) or *Adobe Photoshop* (CS6, Adobe Corp.) unless stated otherwise.

### 4.7 Statistical analysis

Statistical analysis was performed using *Origin Pro* v9.0 (Origin Labs). The mean comparison between the control and treatment groups was analyzed using a *One-Way Analysis of Variance* (ANOVA) with Tukey's HSD post *hoc* test. Data are reported as mean ± s.d. of replicated (*n* = 5) experiments, statistical significance was considered at ∗*p* < 0.05, ∗∗*p* < 0.01, ∗∗∗*p* < 0.001, and ∗∗∗∗*p* < 0.0001. Statistically non-significant data is represented as *n*.*s*.

## CRediT authorship contribution statement

**Sayan Deb Dutta:** Writing – review & editing, Writing – original draft, Validation, Software, Methodology, Investigation, Formal analysis, Data curation, Conceptualization. **Jeong Man An:** Writing – original draft, Software, Resources, Methodology, Investigation, Formal analysis, Data curation. **Jagannath Mondal:** Resources, Methodology, Formal analysis, Data curation. **Subhankar Bose:** Methodology, Formal analysis, Data curation. **Santosh Kumar Jana:** Validation, Methodology, Data curation. **Youjin Seol:** Validation, Formal analysis, Data curation. **Chandan Kuman Maity:** Writing – review & editing, Supervision, Investigation. **Yong-kyu Lee:** Writing – review & editing, Resources, Project administration, Funding acquisition. **Ki-Taek Lim:** Writing – review & editing, Writing – original draft, Supervision, Project administration, Investigation, Funding acquisition.

## Ethical approval and consent to participate

All the animal experiments were approved by the Institutional Review Board (IRB) of the Institute of Animal Care and Use Committee (IACUC) of Korea National University of Transportation Hospital (Permission No. KNUT-2024-A3E).

## Declaration of competing interest

The authors declare that they have no known competing financial interests or personal relationships that could have appeared to influence the work reported in this paper.

## Data Availability

The raw data supporting the findings reported in this study can be obtained from the corresponding author upon reasonable request.
