# Supplementary material for: Ion channel/Stat6-driven nano-immune programming of tissue-resident macrophages by amide-functionalized nanocellulose
Source: Bioact Mater. 2026 Apr 9;62:883–98. doi: 10.1016/j.bioactmat.2026.03.038 (PMC13091342; doi:10.1016/j.bioactmat.2026.03.038)
Supplement: Multimedia component 1 [file mmc1.docx]

**Supplementary Information for:**

**Ion channel/Stat6-driven Nano-Immune Programming of Tissue-resident Macrophages by Amide-functionalized Nanocellulose**

**Contents:**

Figure S1-S13 (Page No. S2-S15)

Supplementary Table S1-S3 (Page No. S16-19)

Supplementary References (Page No. S20)

**
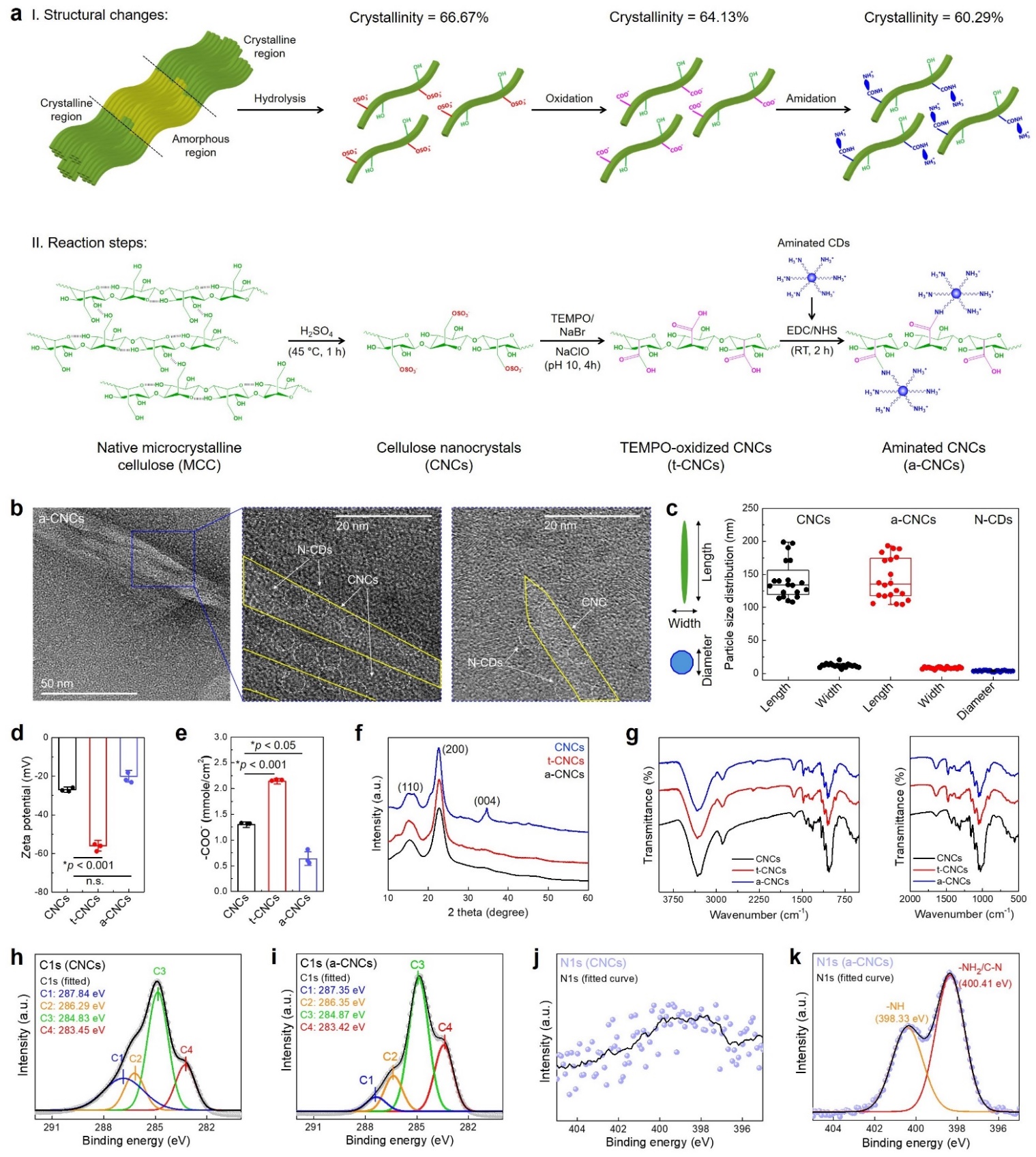
**

**Figure S1. (a)** Schematic illustration of the synthesis steps of a-CNCs from bulk cellulose. **(b)** HR-TEM images showing the distribution of N-CD particles onto the CNCs. **(c)** Size distribution curve of CNCs, a-CNCs, and N-CDs (*n* = 20 counts). **(d)** Zeta potential of the CNCs, t-CNCs, and a-CNCs (*n* = 3). **(e)** Toluidine blue-O test for analyzing the change in surface carboxyl (COO^-^) group densities in CNCs, t-CNCs, and a-CNCs (*n* = 3). **(f)** XRD pattern of the CNCs, t-CNCs, and a-CNCs. **(g)** FT-IR spectra of the CNCs, t-CNCs, and a-CNCs. **(h-k)** High-resolution XPS spectra at the C1s and N1s regions for CNCs and a-CNCs confirm the successful grafting of N-CDs onto CNCs.

**
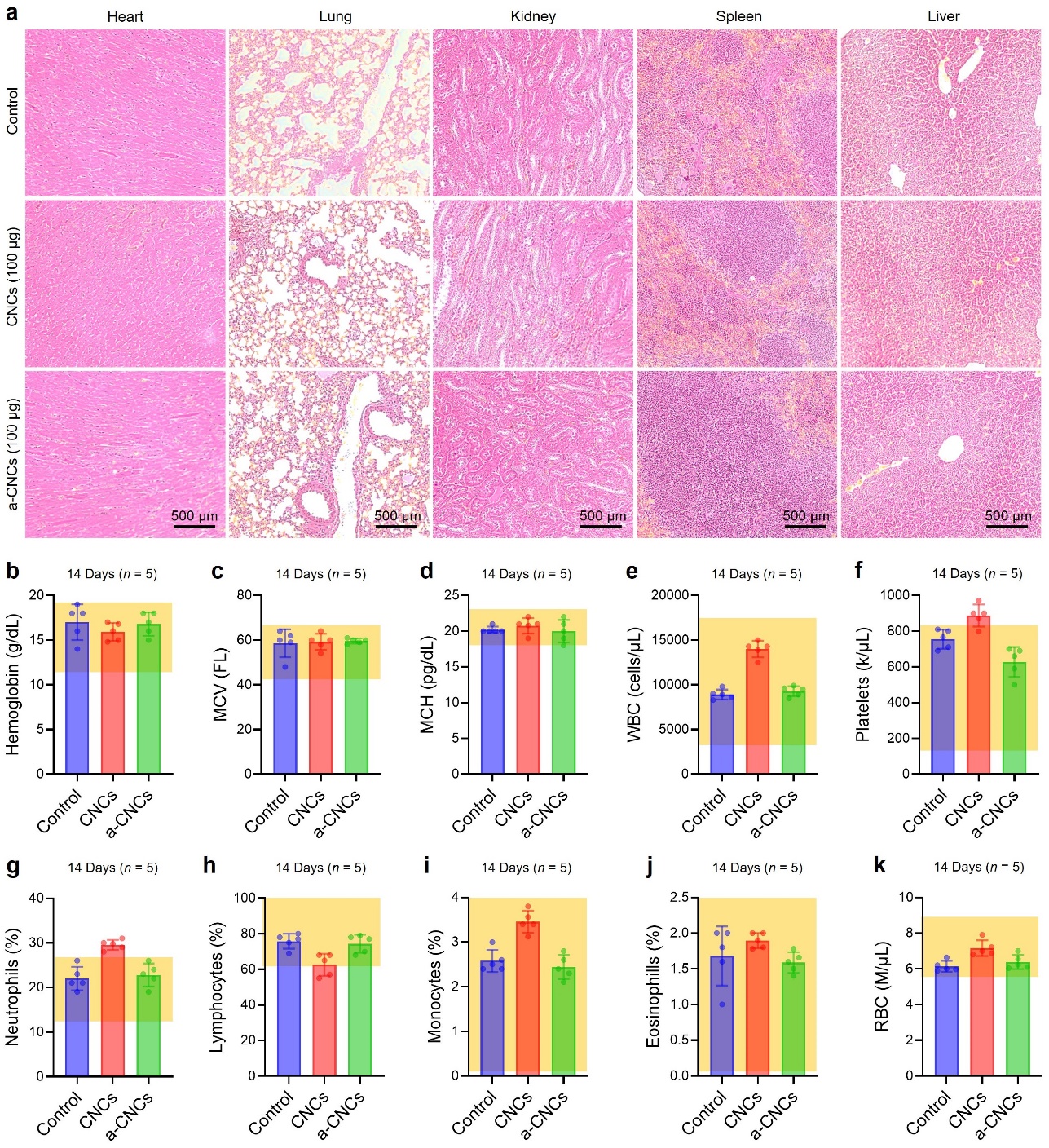
**

**Figure S2.** *In vivo* biocompatibility of pristine CNCs and a-CNCs at day 14. **(a)** Representative H&E staining of major organs (heart, lung, kidney, spleen, and liver) showing the histomorphology after pristine and a-CNCs treatment at day 14. Scale bar: 500 μm. **(b)** Routine blood biochemistry profile of mice after 14 days of administration of pristine CNCs and a-CNCs (100 μg mL^-1^/10 g bw each). Data reported as mean ± s.d. of replicated (*n* = 5) experiments, statistical significance considered at ^*^*p* < 0.05 (One-way ANOVA with Tukey's test *post-hoc* analysis).


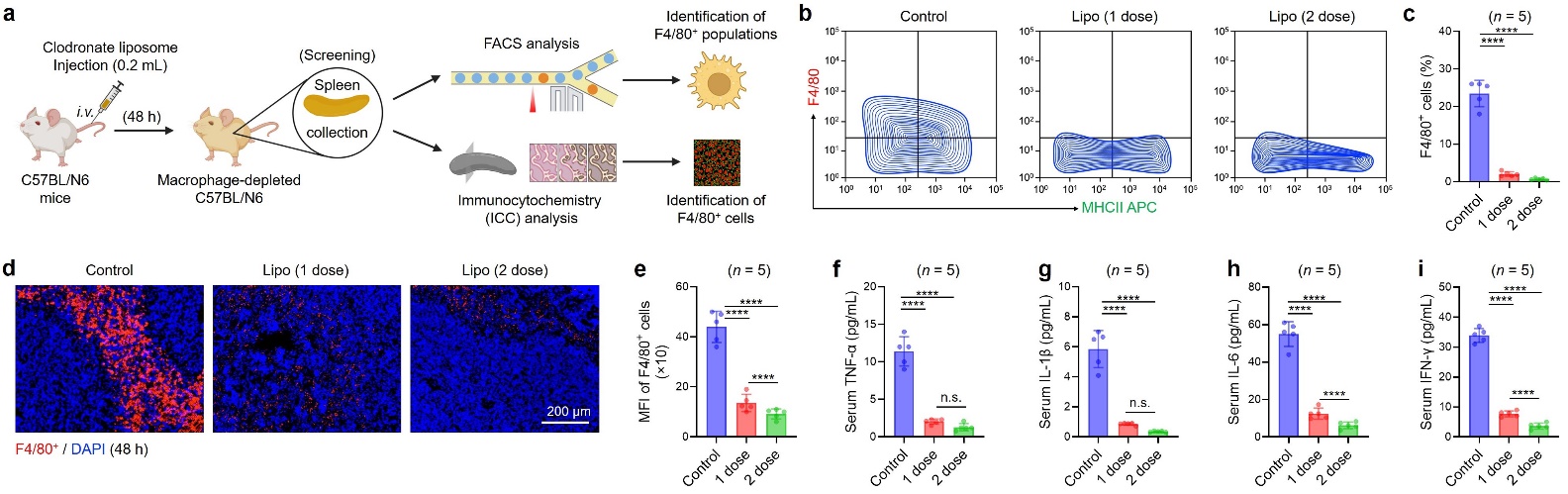


**Figure S3. (a)** Schematic illustration of the macrophage depletion model adopted in this study. **(b)** Representative flow cytometry analysis of mouse spleen macrophage for F4/80 vs. MHC-II after the first (24 h) and second (48 h) dose of liposome treatment. **(c)** Statistical analysis of F4/80^+^ cells in the spleen-derived macrophages (*n* = 5 each). **(d)** Immunostaining of F4/80 (red) showing the effect of liposome treatment on spleen tissue. Scale bar: 200 μm. **(e)** Statistical analysis of F4/80^+^ cells in spleen tissue (*n* = 5 each). **(f-i)** Estimation of serum TNF-α, IL-1β, IL-6, and IFN-γ content after liposome tretament. Data reported as mean ± s.d. of replicated (*n* = 5 each) experiments, statistical significance considered at ^****^*p* < 0.0001 (One-way ANOVA with Tukey's test *post-hoc* analysis).


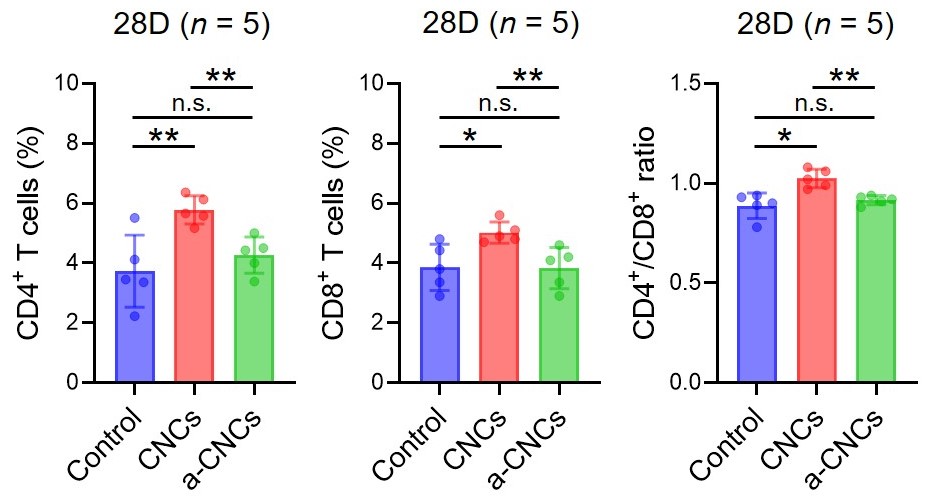


Figure S4. Statistical analysis of CD4^+^/CD8^+^ cells in the spleen tissue after 28-days post-administration of pristine CNCs and a-CNCs. Data reported as mean ± s.d. of replicated (*n* = 5) experiments, statistical significance considered at ^*^*p* < 0.05 and ^**^*p* < 0.01 (One-way ANOVA with Tukey's test *post-hoc* analysis).

**
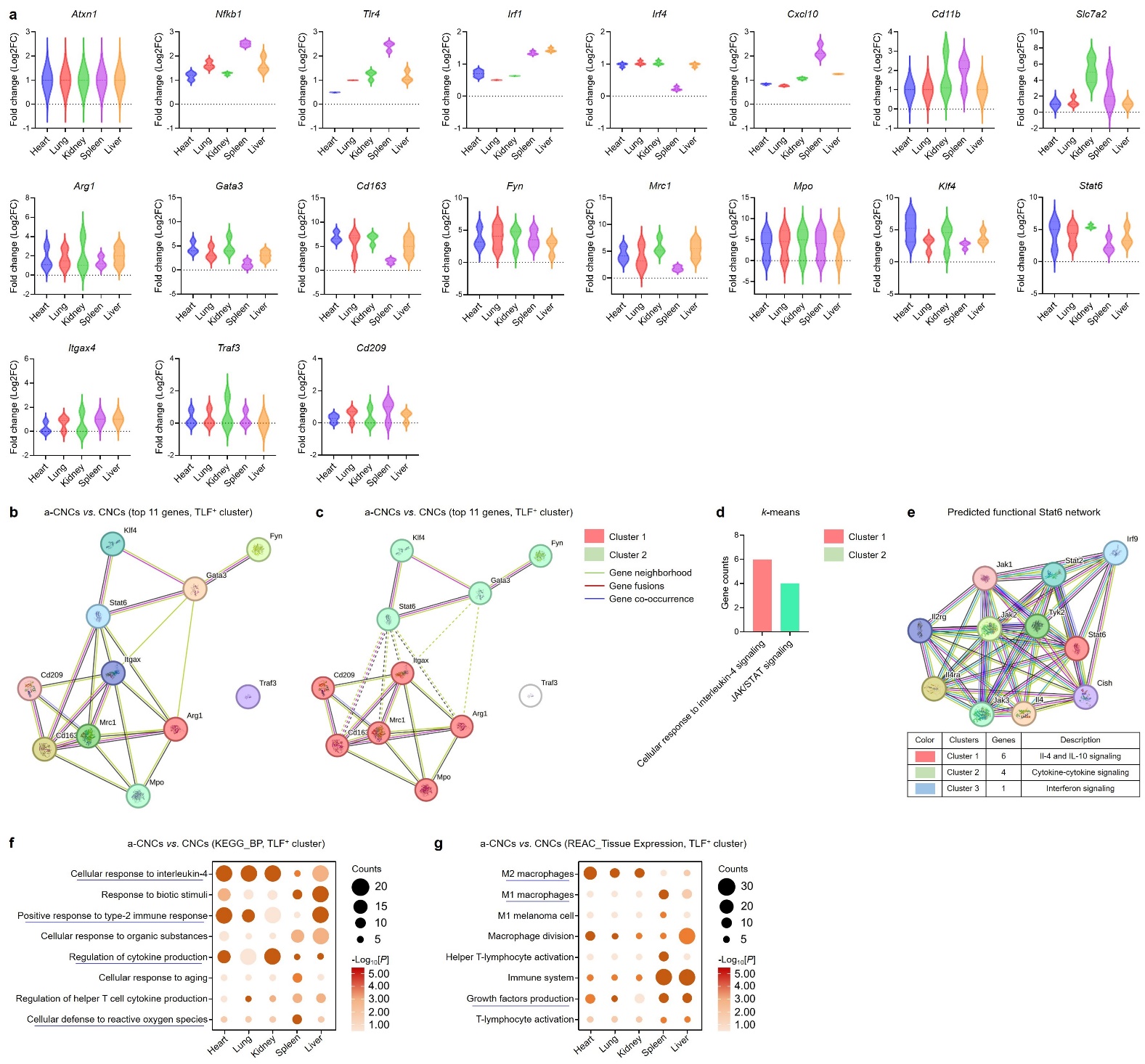
**

**Figure S5.** Bioinformatics analysis of single-cell transcriptomics (scRNA-Seq) data for TLF^+^ clusters. **(a)** Expression changes of the top 19 genes involved in macrophage differentiation were identified from the TLF^+^ cluster of all organs at day 14. **(b)** STRING protein-protein interaction study of the top 19 DEGs. **(c, d)** *k*-means clustering results of the shared network of 19 key genes from the TLF^+^ cluster. **(e)** Representative functional network predicted for *Stat6*, a kety gene differentially expressed in TLF^+^ cluster. **(f, g)** KEGG (KEGG_BP) gene and Reactome (REAC_Tissue Expression) pathway enrichment analysis showing the activation of top regulatory pathways when comparing a-CNCs vs. CNCs at day 14 for the TLF^+^ cluster.


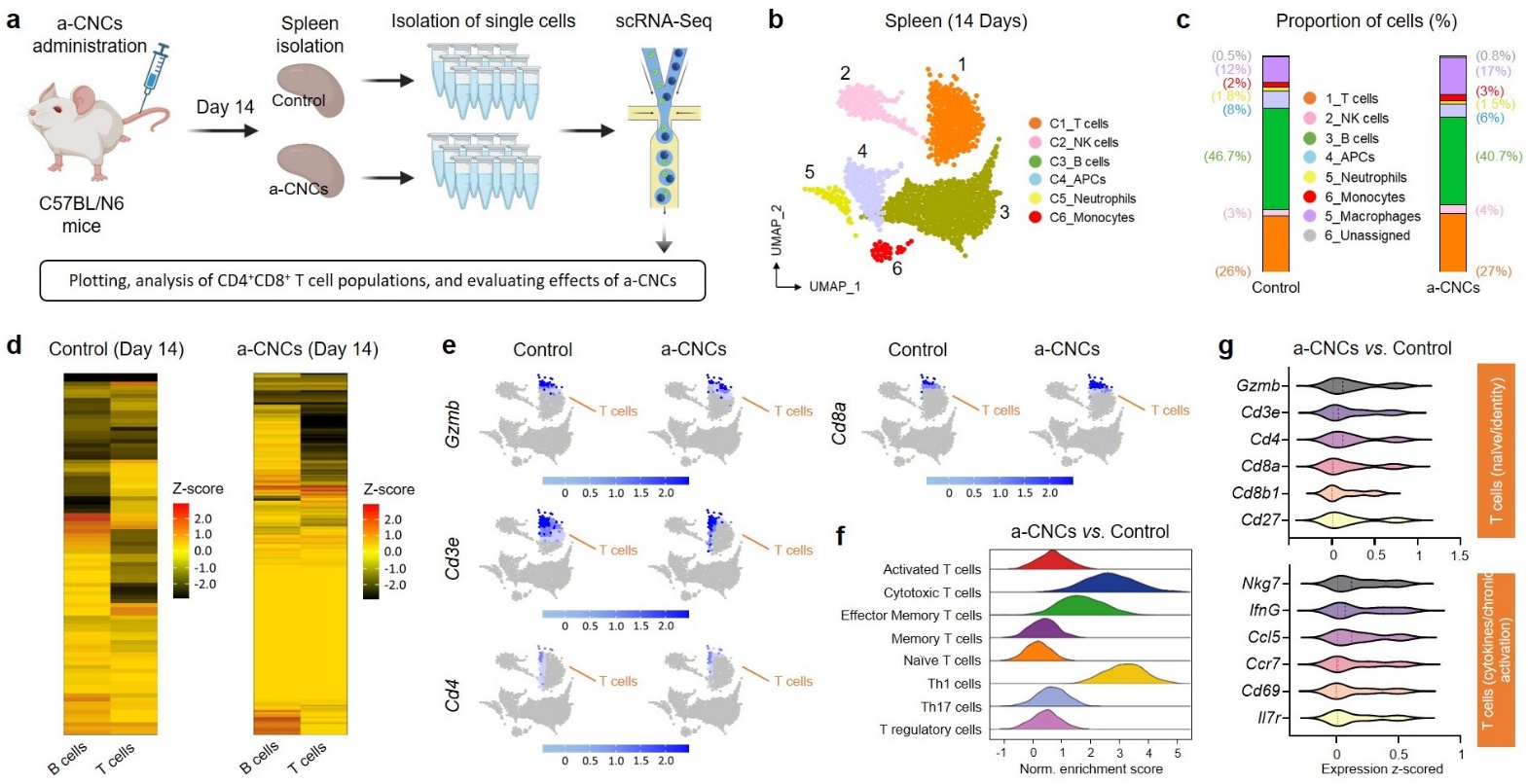


**Figure S6.** Single-cell transcriptomic (scRNA-Seq) analysis reveals T cell state alterations following a-CNCs administration at day 14. **(a)** Schematic illustration of the experimental workflow. C57BL/6 mice received a-CNCs, spleens were harvested at Day 14, single cells were isolated and subjected to scRNA-seq, followed by analysis of CD4⁺ and CD8⁺ T-cell populations and evaluation of a-CNC-induced changes. **(b)** UMAP visualization of splenic immune cells colored by cell type annotation, identifying T cells (C1), NK cells (C2), B cells (C3), APCs (C4), neutrophils (C5), and monocytes (C6). **(c)** Proportion of major immune cell populations in control versus a-CNC-treated mice, showing treatment-associated shifts in immune composition. **(d)** Heatmaps showing scaled expression (Z-score) of B and T cell–associated genes in the control and a-CNC conditions. **(e)** UMAP feature plots showing the expression of representative T-cell markers (**Gzmb, Cd4, Cd8a, Cd8b**) in control and a-CNCs group. **(f)** Ridge plot analysis of normalized enrichment scores for Eomes transcription factor binding across T-cell subsets. **(g)** differential expression (Z-scored) of effector and memory-associated genes between a-CNC and control groups.


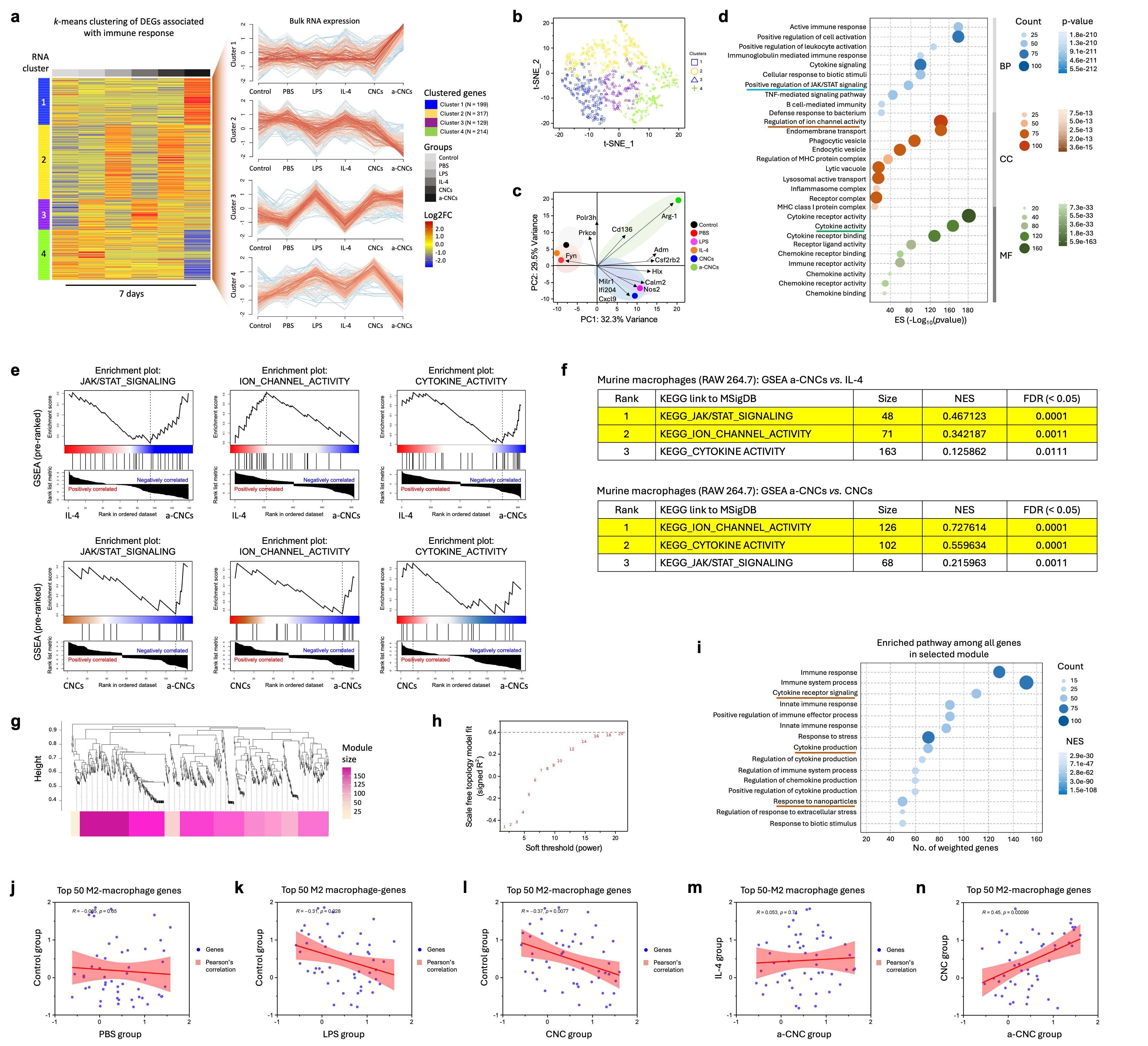


**Figure S7.** a-CNCs treatment stimulates M2 macrophage polarization via global immune regulation. **(a)** k-means hierarchical clustering of DEGs associated with the immune response. Graphical representation of bulk RNA expression (Log2 FC) in various clusters (1-4) following PBS, LPS, IL-4, CNCs, and a-CNCs treatment for 14 days. **(b)** t-SNE maps showing the distribution of genes in various clusters. **(c)** Principal component analysis (PCA) of various groups and their corresponding clustered genes. Insets are key regulatory genes that are correlated across various treatment groups. **(d)** GO enrichment analysis of top-upregulated (Log FC > 2, FDR < 0.05) genes found in RNA-Seq of RAW 264.7 cells after 14 days of culture in a-CNC group. The activation of M2 polarization pathway terms, including JAK/STAT signaling, ion channel activity, and cytokine activity, was highly upregulated following a-CNC treatment. **(e)** Gene set enrichment analysis (GSEA) plots showing a high correlation for JAK/STAT signaling, ion channel activity, and cytokine activity when comparing IL-4, CNCs, and a-CNCs from RNA-Seq data. These gene sets were found to be associated with M2-type macrophage polarization. **(f)** Demonstration of top-ranked GSEA KEGG terms in RAW 264.7 cells showing that a-CNCs treatment induced the M2 polarization via inducing the voltage-gated ion channels and higher cytokine secretion. **(g)** The WGCNA results show the gene dendrogram obtained by average linkage hierarchical clustering among various groups. **(h)** Scale independence of WGCNA power selection. **(i)** Analysis of pathway enrichment among all genes in WGCNA through KEGG. **(j-n)** Pearson's correlation analysis of the top 50 genes related to M2-type macrophage polarization in various groups after 14 days of treatment.


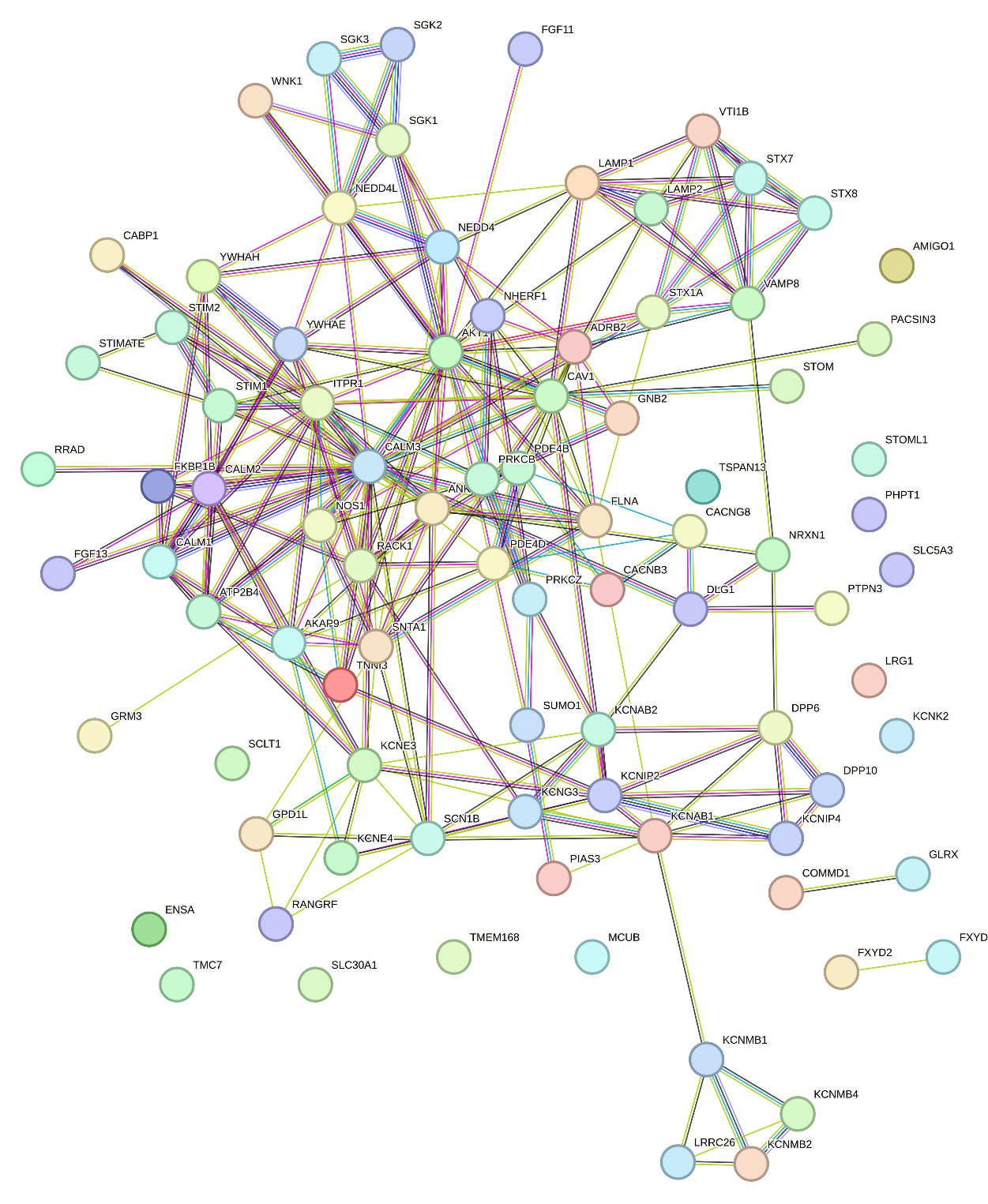


**Figure S8.** STRING protein-protein interaction study of the key DEGs associated with ion channel activity in RAW 264.7 cells when comparing the a-CNCs *vs*. CNCs group at day 14.

**
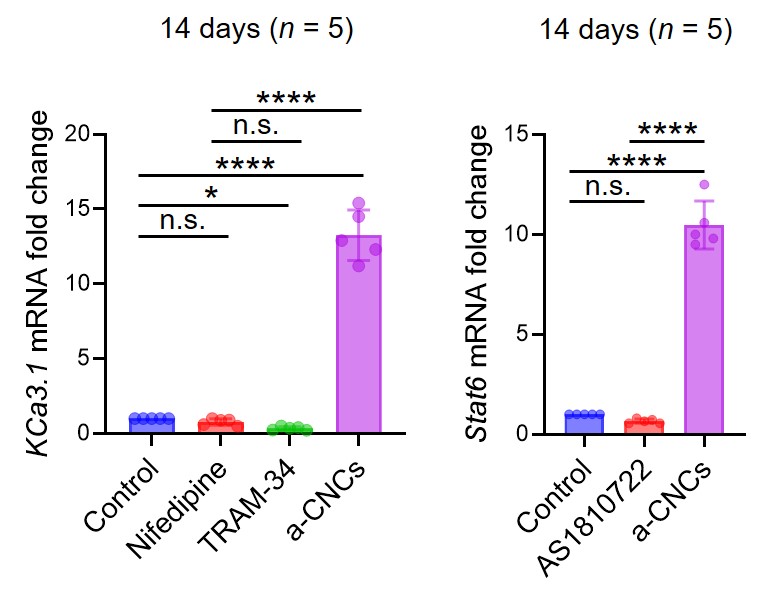
**

**Figure S9.** qRT-PCR analysis of RAW 264.7 cells showing the expression of *KCa3.1* and *Stat6* gene markers expression at day 14 following pharmacological inhibition. Data reported as mean ± s.d. of replicated (*n* = 5) experiments, statistical significance considered at ^*^*p* < 0.05 and ^****^*p* < 0.0001 (One-way ANOVA with Tukey's test *post-hoc* analysis).


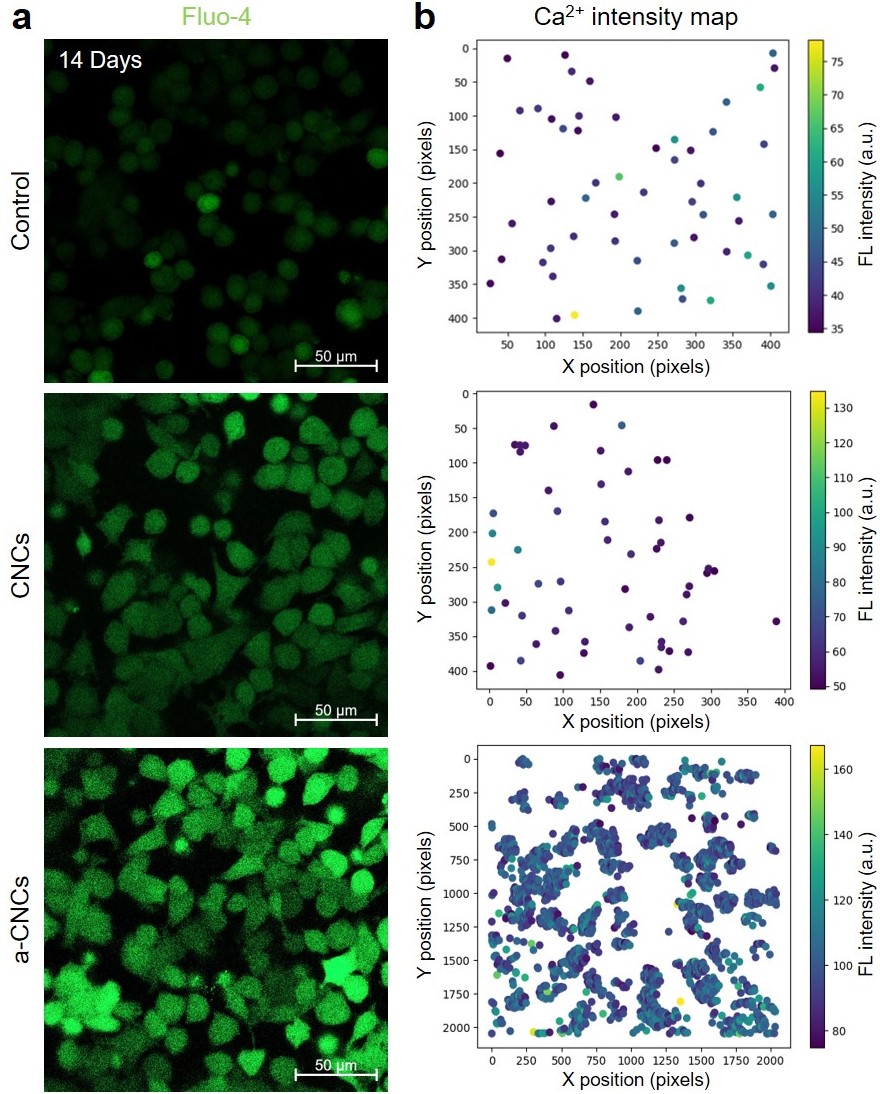


**Figure S10.** Live cell Ca^2+^ imaging of RAW 264.7 cells at day 14. **(a)** Representative CLSM images of RAW 264.7 cells stained with Fluo-4 AM at day 14. Scale bar: 50 μm. **(b)** Cell-based Ca^2+^ intensity heat maps showing the spatial Ca^2+^ spikes in each cells (*n* = 35-60).


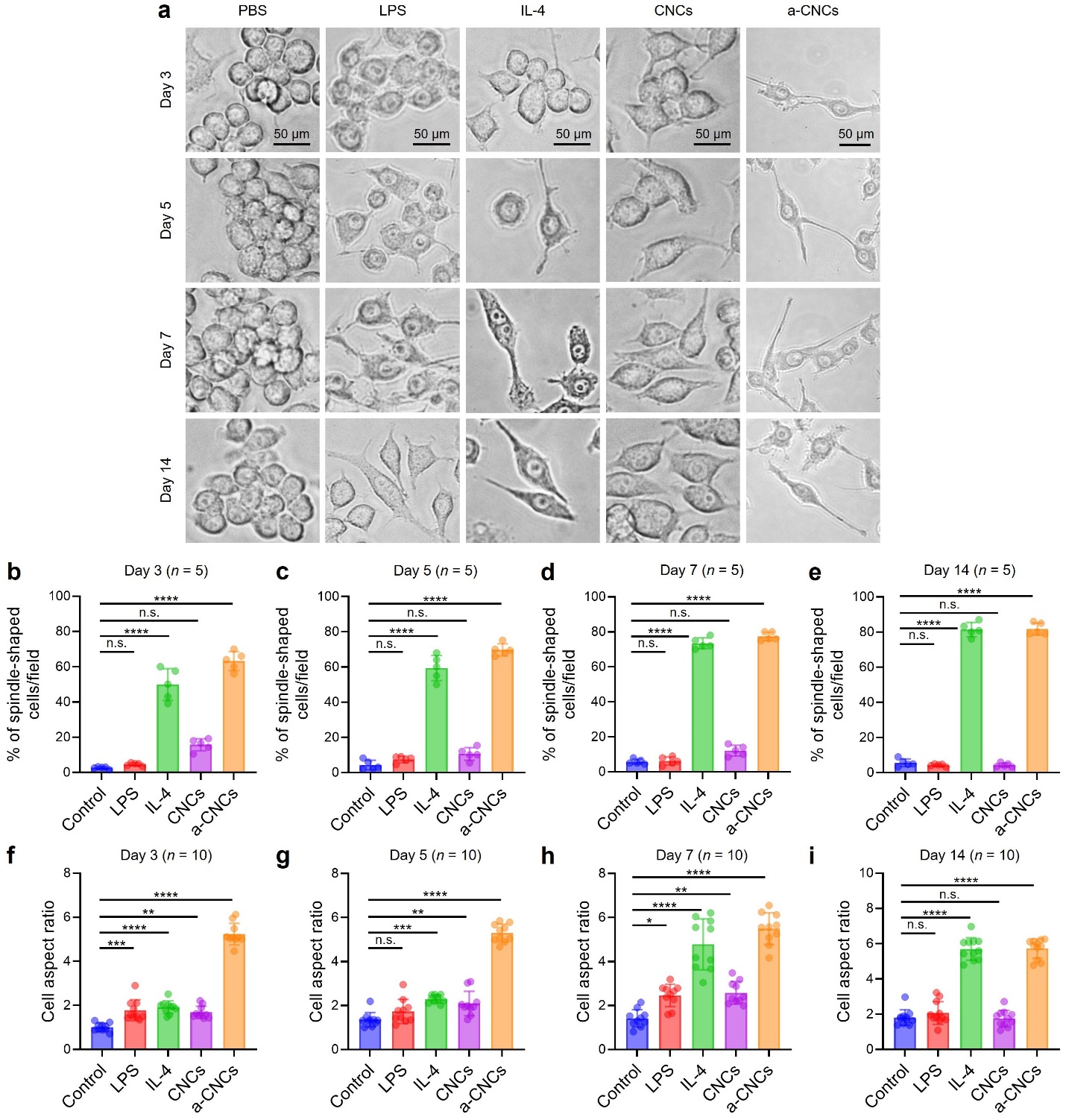


**Figure S11.** *In vitro* M1/M2 macrophage polarization potential of CNCs and a-CNCs on RAW 264.7 cells. **(a)** Representative bright field images of RAW 264.7 cells showing the change in morphology in various formulations at the indicated time points. Scale bar: 50 μm. **(b-e)** Statistical analysis of the % of spindle-shaped (=elongated) cells in various formulations (*n* = 5 each). **(f-i)** Calculation of cell aspect ratio (=long axis/short axis) of RAW 264.7 cells in various groups (*n* = 10 each). Data reported as mean ± s.d. of replicated experiments, statistical significance considered at ^*^*p* < 0.05, ^**^*p* < 0.01, ^***^*p* < 0.001, and ^****^*p* < 0.0001 (One-way ANOVA with Tukey's test *post-hoc* analysis).


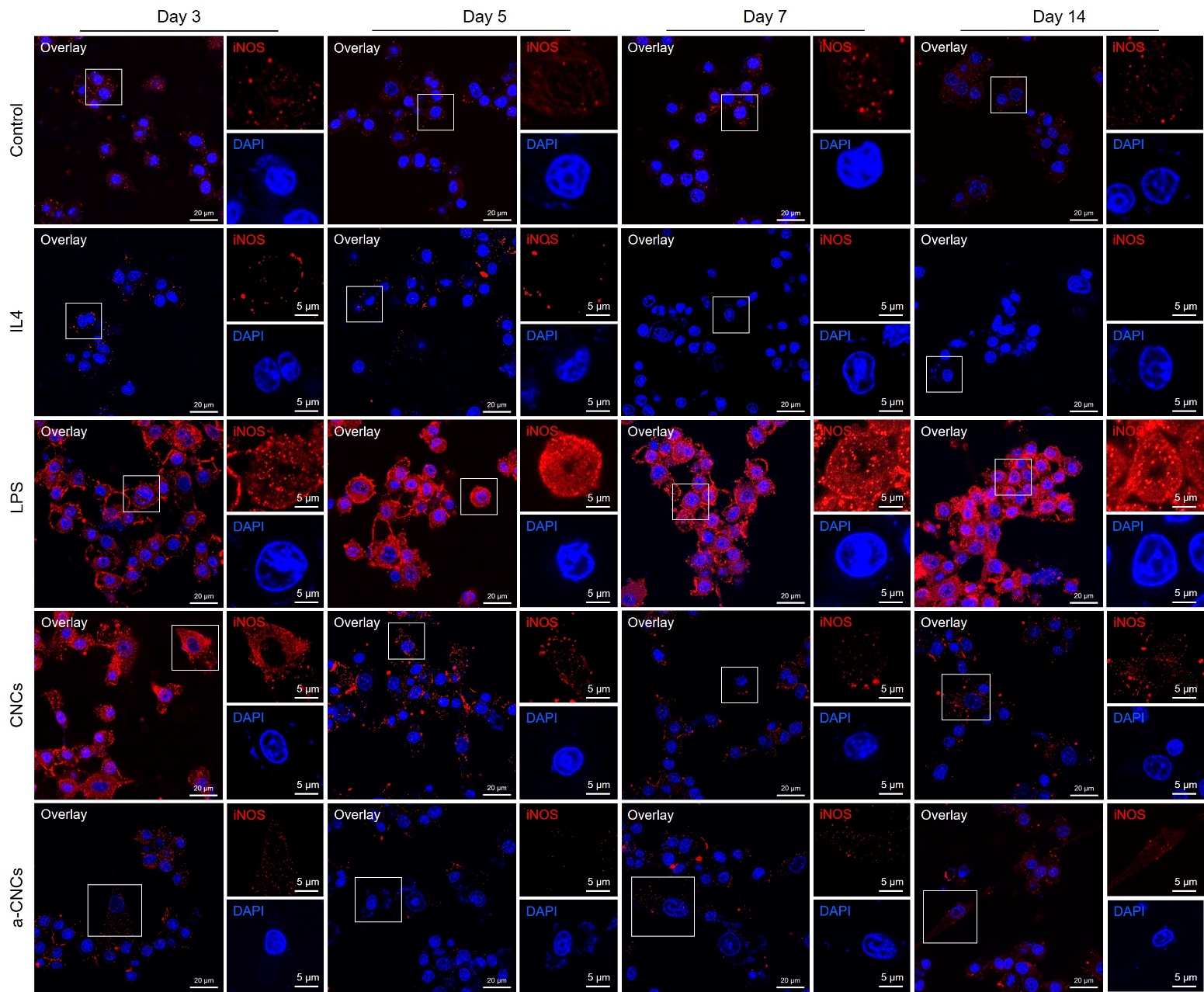


**Figure S12.** Immunostaining images of RAW 264.7 cells showing the expression of Nos2 (red) in various groups up to 14 days. Scale bar: 5 and 20 μm.

**
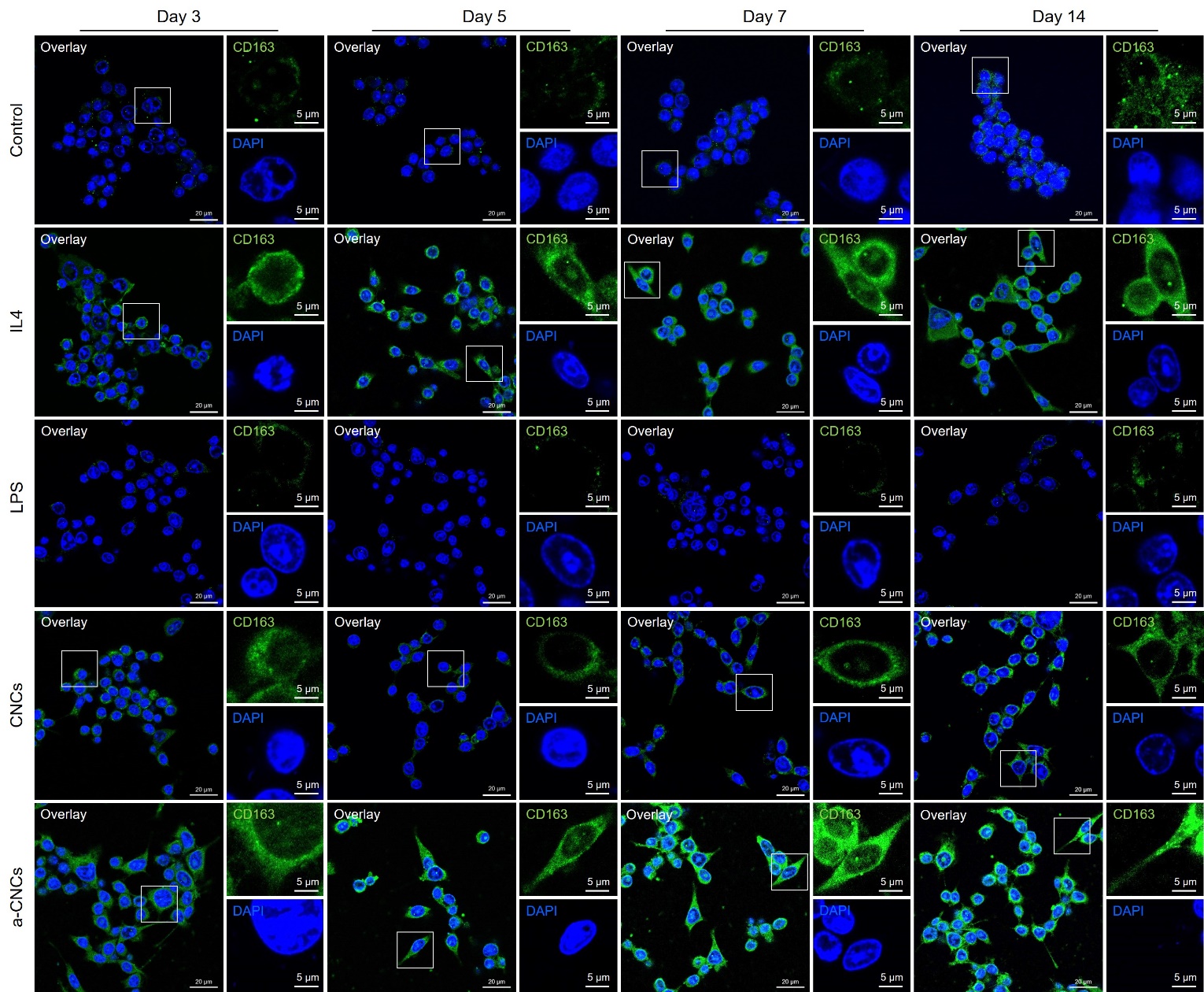
**

**Figure S13.** Immunostaining images of RAW 264.7 cells showing the expression of Cd163 (green) in various groups up to 14 days. Scale bar: 5 and 20 μm.

**Supplementary Table S1.** A comparative study of various functionalized nanocellulose and its derivatives used for macrophage phenotyping reported in the literature.

| **Material/size** | **Macrophage**  **fate** | ***In vitro/in vivo* effects** | **Study/assay**  **time** | **References** |
| --- | --- | --- | --- | --- |
| CNCs^1^  (~135-150 nm) | M1 activation | *In vitro* activation of  IFNG, IL1b, IL6, Nos2, TNF-α factors; no *in vivo* study | 24-48 h (*in*  *vitro*) | [1] |
| CNCs^1^  (~150-720 nm) | M1 activation | *In vitro* activation of IL-1β;  no *in vivo* study | 24 h (*in vitro*) | [2] |
| CNFs^1^  (~6700 nm) | M1 activation | *In vitro* activation of IL-1β;  no *in vivo* study | 24 h (*in vitro*) | [2] |
| CNCs^1^  (~140 nm) | M2 polarization at  Day 1; M1  polarization at  day 3 | *In vitro* activation of Nos2, CD68,  CD86, IL1R1, CCL3, CCL4, and  CXCL16; no *in vivo* study | 72 h (*in vitro*) | [3] |
| CNPs^2^  (~43 nm) | M2 polarization at  Day 1; M1  polarization at  day 3 | *In vitro* activation of Nos2, CD68,  CD86, IL1R1, CCL3, CCL4, and  CXCL16; no *in vivo* study | 72 h (*in vitro*) | [3] |
| BNC^3^ | M2 polarization | No *in vitro* direct evidence of  markers expression, confirmed  based on morphology; no *in*  *vivo* study | 5 days (*in*  *vitro*) | [4] |
| HPCNF^4a^  (~34 nm fiber  diameter) | M1 activation  (LPS challenged) | *In vitro* activation of TNF-α and  IL-1β; no *in vivo* study | 24 h (*in vitro*) | [5] |
| HPTACNF^4b^  (~24 nm fiber  diameter) | M2 activation  (LPS challenged) | *In vitro* activation of IL-10 and  CD206; no *in vivo* study | 24 h (*in vitro*) | [5] |
| CNCs^5^  (~25-80 nm fiber  diameter) | M1 activation by  anionic CNCs,  M2 activation by  cationic CNCs  (LPS challenged) | *In vitro* activation of TNF-α and  IL-1ra by THP-1 cells when  Exposed to anionic CNCs; no *in*  *vivo* study | 24 h (*in vitro*) | [6] |
| CNC gel  (~137-149 nm) | ROS-induced  toxicity; M1  activation | *In vitro* activation of IL-6, IL-8,  and MCP-1; no *in vivo* evidence  of inflammation | 72 h (*in vitro*) | [7] |
| P-CNFs^6^  (~16 nm fiber  diameter) | M2 polarization | *In vitro* activation of CD206 and CD14 markers; no *in vivo* study | 6 days | [8] |
| CNC-OSO_3_^-^  (~120 nm) | Inert activity, no  Significant M1/M2  activation | *In vitro* negative response to  TLR4 activity after 24 h; good  *in vivo* biocompatibility and low  level of TNF-α/IL-6 production  at day 30; no systemic *in vivo*  organ-specific polarization study | 24 h (*in vitro*)  2-30 days (*in*  *vivo*) | [9] |
| TEMPO-CNCs  scaffold | M1 activation at  day 3 | *In vitro* pro-inflammatory  Cytokines/chemokines secretion  By U937 cells; *in vivo* pro-  inflammatory activation at day 4,  While downregulation at day 30;  No clear demonstration of M1/  M2 states; no organ-wide  analysis | 3 days (*in*  *vitro*)  30 days (*in*  *vivo*) | [10] |
| CNCs/CNFs  (~105-110 nm) | Mild inflammatory  response in lungs | CNCs-induced pro-inflammatory  Activation of IL-1α, TNF-α,  IL-1β; no upregulation of T cell  populations (helper & killer T  cells); mild upregulation of NK  cells up to 28 days *in vivo*, no  study on organ-wide and systemic  macrophage polarization *in vivo* | 24 h (*in vitro*)  28 days (*in*  *vivo*) | [11] |
| 3D BNC  Scaffold^7^ | N/A | Good *ex vivo* blood  biocompatibility;  No direct evidence of M1/M2  polarization *in vitro* and *in vivo* | 90 days (*ex*  *vivo*) | [12] |
| Oxalate-modified  CNCs | N/A | Good *in vivo* blood compatibility  and no oxidative stress response  in major organs; high expression  of Nos2^+^ cells in the liver tissue  (mild hepatotoxicity); No  systemic M1/M2 polarization  study *in vivo* | 7 days (*in*  *vivo*) | [13] |
| **Amine-modified**  **CNCs (N-CDs**  **functionalized**  **CNCs or**  **a-CNCs)** | ***In vitro* and *in vivo***  **M2 macrophage**  **polarization** | **Good biocompatibility, a-CNCs**  **-induced M2 polarization of**  **RAW 264.7 cells in vitro up to 14 days; *in vivo* organ-specific M2**  **polarization via Stat6-ion**  **channel enhancement; *in vivo* macrophage maturation, static developmental trajectories and**  **no detectable adaptive**  **immunity up to 28 days (multi-**  **omics and pharmacological**  **approach)** | **-14 days (*in***  ***vitro*,**  **subacute**  **stage)**  **-14-28 days**  **(*in vivo*,**  **subacute**  **to pro-**  **healing**  **stage)** | **This study** |

^1^hydroxyl terminated (rod-shaped), ^2^hydroxyl terminated (spherical-shaped), ^3^bacterial nanocellulose (fibrous mesh), ^4a^carboxymethyl CNF (fibrous mesh), ^4b^hydroxypropyltrimethylammonium-modified CNF (fibrous mesh), ^5^*Cladophora* algae-derived cationic and anionic films (fiber mesh), ^6^phosphate-modified CNFs (fiber mesh), ^7^3D BNC scaffold derived from *Komagataeibacter medellinensis* bacteria, ROS: reactive oxygen species.

**Supplementary Table S2.** List of primary and secondary antibodies used in this study.

| **Antibody** | **Type** | **Catalogue No.** | **Company** |
| --- | --- | --- | --- |
| Anti-iNOS (=Nos2) | Primary | #sc-7271 | Santa Cruz Biotechnology |
| Anti-Cd163 | Primary | #sc-58965 | Santa Cruz Biotechnology |
| Anti-KCa3.1 | Primary | #PA5-142531 | Thermo-Fischer Scientific |
| Anti-Scn1b | Primary | #PA5-41003 | Thermo-Fischer Scientific |
| Anti-F4/80 | Primary | #sc-377009 | Santa Cruz Biotechnology |
| Anti-CD3 | Primary | #ab-11089 | Abcam |
| Anti-CD4 | Primary | #ab-133616 | Abcam |
| Anti-CD8 | Primary | #ab-316778 | Abcam |
| Anti-CD4 AF488 | Secondary | #ab-196372 | Abcam |
| Anti-CD8 AF647 | Secondary | #ab-237365 | Abcam |
| AF594 | Secondary | #sc-7271 AF594 | Santa Cruz Biotechnology |
| m-IgG*k* BP-FITC | Secondary | #sc-516140 | Santa Cruz Biotechnology |
| Goat anti-Rabbit IgG AF594 | Secondary | #A-11012 | Thermo-Fischer Scientific |
| Goat anti-Rabbit IgG AF405 | Secondary | #A-31556 | Thermo-Fischer Scientific |

**Supplementary Table S3.** List of primary and secondary antibodies used in this study.

| **Gene(s)** | **Forward sequence (5' to 3')** | **Reverse sequence (5' to 3')** |
| --- | --- | --- |
| *Il-1ra* | AGTACCCTCAGAGCTCCCAG | AGGGTGCCCAGACTCTCTAG |
| *Il-1β* | CTAGGTTGGGCTTGGGAGTG | CCCATGTTGTAGTGACCCCC |
| *Tlr4* | TTCTCCTCGGCCTGTCCATA | TGGTGTCTGGTGTGCTTTGT |
| *Nos2* | GCACATCAAAGCGGCCATAG | CGGCAAACATGACTTCAGGC |
| *Il-6* | CAAGGAGCTGAAGGGGTCTG | CCAGGCCTACACTCCCTACT |
| *Cd11b* | GAAAGGGGGCATAGCAAGGT | CTCTGCCACCCATTTCCCAT |
| *Nfkb* | GTGGTAGGAAGGGGAAAGCC | CCAGGCATGGGAAGTGGATT |
| *Tnf-a* | CCCTCACACTCAGATCATCTTCT | GCTACGACGTGGGCTACAG |
| *Arg-1* | AACACGGCAGTGGCTTTAAC | GTCAGTCCCTGGCTTATGGTT |
| *Mrc1* | CGGACATGGCTGGTAAAGGT | TTGGTTCATGCCCTAGCTGG |
| *Tgf-β1* | CCCCAAGCAAAACACTCGTG | CAGAGGAAACTGGCTGGAGG |
| *Cd206* | CAGAACTGTGAGGCCAGGAG | AAATGGCTTCCTGGAGAGCC |
| *Cd163* | GTGGTCAACTCCGCTTGGTA | CTTGGGGCACCATCTGTGAT |
| *Stat6* | TTCTTGGGAGCCTCACTGAAAG | AGCCGTTGCAGTTTTTCTGG |
| *Il-10* | GCTCTTACTGACTGGCATGAG | CGCAGCTCTAGGAGCATGTG |
| *Vegfa* | TTTGCTGTCACTGCCGTTTG | CCGCAGCAATCCATCCTAAAAC |
| *Gapdh* | CATACAGGTTTCTCCAGGCG | TTGTGATGGGTGTGAACCAC |

**Supplementary References**

[1] J.S. Erdem, M. Alswady-Hoff, T.K. Ervik, Ø. Skare, D.G. Ellingsen, S. Zienolddiny, Cellulose nanocrystals modulate alveolar macrophage phenotype and phagocytic function, *Biomaterials* 203 (2019) 31–42.

[2] J. Li, X. Wang, C.H. Chang, J. Jiang, Q. Liu, X. Liu, Y.P. Liao, T. Ma, H. Meng, T. Xia, Nanocellulose length determines the differential cytotoxic effects and inflammatory responses in macrophages and hepatocytes, *Small* 17(38) (2021) 2102545.

[3] D.K. Patel, K. Ganguly, S.D. Dutta, T.V. Patil, K.-T. Lim, Cellulose nanocrystals vs. cellulose nanospheres: A comparative study of cytotoxicity and macrophage polarization potential, *Carbohydrate Polymers* 303 (2023) 120464.

[4] K.P.V. de Oliveira, M.Y. Yitayew, A.P.A. Bastos, S.C.N. Mandrik, L.M. Porto, M. Tabrizian, Transparent 3-layered bacterial nanocellulose as a multicompartment and biomimetic scaffold for co-culturing cells, *Journal of Functional Biomaterials* 16(6) (2025) 208.

[5] V.R. Lopes, C. Sanchez-Martinez, M. Strømme, N. Ferraz, In vitro biological responses to nanofibrillated cellulose by human dermal, lung and immune cells: Surface chemistry aspect, *Particle and fibre toxicology* 14(1) (2017) 1.

[6] K. Hua, M. Strømme, A. Mihranyan, N. Ferraz, Nanocellulose from green algae modulates the in vitro inflammatory response of monocytes/macrophages, *Cellulose* 22(6) (2015) 3673–3688.

[7] A.L. Menas, N. Yanamala, M.T. Farcas, M. Russo, S. Friend, P.M. Fournier, A. Star, I. Iavicoli, G.V. Shurin, U.B. Vogel, Fibrillar vs crystalline nanocellulose pulmonary epithelial cell responses: Cytotoxicity or inflammation?, *Chemosphere* 171 (2017) 671–680.

[8] M. Heilala, R. Turpin, N. Pahimanolis, Nonappa, O. Ikkala, J. Klefström, P.M. Munne, Nanofibrillar cellulose hydrogels with anionic surface modifications for modulating macrophage phenotype in 3D culture, *ACS applied materials & interfaces* 17(28) (2025) 40082–40100.

[9] A.M. Weiss, N. Macke, Y. Zhang, C. Calvino, A.P. Esser-Kahn, S.J. Rowan, In vitro and in vivo analyses of the effects of source, length, and charge on the cytotoxicity and immunocompatibility of cellulose nanocrystals, *ACS biomaterials science & engineering* 7(4) (2021) 1450–1461.

[10] A. Rashad, S. Suliman, M. Mustafa, T.Ø. Pedersen, E. Campodoni, M. Sandri, K. Syverud, K. Mustafa, Inflammatory responses and tissue reactions to wood-Based nanocellulose scaffolds, *Materials Science and Engineering: C* 97 (2019) 208–221.

[11] K. Fujita, S. Obara, J. Maru, Y. Kawai, S. Endoh, A. Moriyama, Pulmonary inflammation and immune responses induced by nanocellulose: Insights from in vivo and in vitro models, *Current Research in Toxicology* (2025) 100259.

[12] M. Osorio, A. Cañas, J. Puerta, L. Díaz, T. Naranjo, I. Ortiz, C. Castro, Ex vivo and in vivo biocompatibility assessment (blood and tissue) of three-dimensional bacterial nanocellulose biomaterials for soft tissue implants, *Scientific reports* 9(1) (2019) 10553.

[13] C. Otuechere, A. Adewuyi, O. Adebayo, I. Ebigwei, In vivo hepatotoxicity of chemically modified nanocellulose in rats, *Human & Experimental Toxicology* 39(2) (2020) 212–223.
